# Cerebellar Calcium-Binding Protein and Neurotrophin Receptor Defects in Down Syndrome and Alzheimer's Disease

**DOI:** 10.3389/fnagi.2021.645334

**Published:** 2021-03-12

**Authors:** Jennifer C. Miguel, Sylvia E. Perez, Michael Malek-Ahmadi, Elliott J. Mufson

**Affiliations:** ^1^Department of Neurobiology, Barrow Neurological Institute, Phoenix, AZ, United States; ^2^Department of Biomedical Informatics, Banner Alzheimer's Institute, Phoenix, AZ, United States; ^3^Department of Neurology, Barrow Neurological Institute, Phoenix, AZ, United States

**Keywords:** Down syndrome, Alzheimer's disease, cerebellum, calcium binding proteins, Purkinje cells, nerve growth factor receptors, amyloid, tau

## Abstract

Cerebellar hypoplasia is a major characteristic of the Down syndrome (DS) brain. However, the consequences of trisomy upon cerebellar Purkinje cells (PC) and interneurons in DS are unclear. The present study performed a quantitative and qualitative analysis of cerebellar neurons immunostained with antibodies against calbindin D-28k (Calb), parvalbumin (Parv), and calretinin (Calr), phosphorylated and non-phosphorylated intermediate neurofilaments (SMI-34 and SMI-32), and high (TrkA) and low (p75^NTR^) affinity nerve growth factor (NGF) receptors as well as tau and amyloid in DS (*n* = 12), Alzheimer's disease (AD) (*n* = 10), and healthy non-dementia control (HC) (*n* = 8) cases. Our findings revealed higher Aβ_42_ plaque load in DS compared to AD and HC but no differences in APP/Aβ plaque load between HC, AD, and DS. The cerebellar cortex neither displayed Aβ_40_ containing plaques nor pathologic phosphorylated tau in any of the cases examined. The number and optical density (OD) measurements of Calb immunoreactive (-ir) PC soma and dendrites were similar between groups, while the number of PCs positive for Parv and SMI-32 were significantly reduced in AD and DS compared to HC. By contrast, the number of SMI-34-ir PC dystrophic axonal swellings, termed torpedoes, was significantly greater in AD compared to DS. No differences in SMI-32- and Parv-ir PC OD measurements were observed between groups. Conversely, total number of Parv- (stellate/basket) and Calr (Lugaro, brush, and Golgi)-positive interneurons were significantly reduced in DS compared to AD and HC. A strong negative correlation was found between counts for Parv-ir interneurons, Calr-ir Golgi and brush cells, and Aβ_42_ plaque load. Number of TrkA and p75^NTR^ positive PCs were reduced in AD compared to HC. These findings suggest that disturbances in calcium binding proteins play a critical role in cerebellar neuronal dysfunction in adults with DS.

## Introduction

Down Syndrome (DS), or trisomy 21, is a genetic disorder caused by an extra copy of chromosome 21 (HSA21), which is characterized by developmental delay and intellectual disability (Potier and Reeves, 2016). By the age of 40, people with DS develop a pathological profile consisting of amyloid plaques and tau containing neurofibrillary tangles (NFTs) within the neo and limbic cortex similar to Alzheimer's disease (AD) (Wisniewski et al., 1985; Davidson et al., 2018) as well as reduction in brain volume (de la Monte and Hedley-Whyte, 1990; Weis et al., 1991; Kesslak et al., 1994; Aylward et al., 1999; Teipel et al., 2003; Edgin et al., 2015; Cipriani et al., 2018), including the cerebellum (Weis et al., 1991; Jernigan et al., 1993). In DS, cerebellar hypoplasia has been attributed to developmental disturbances in neurogenesis (Guidi et al., 2011) that contribute to deficits in cognition (Jernigan et al., 1993; Pinter et al., 2001; Pennington et al., 2003; Carr, 2005; Lott and Dierssen, 2010) and motor function (Spanò et al., 1999; Lee et al., 2020). Although cerebellar atrophy and beta amyloid plaques, but not NFTs, develop during aging in DS (Mann and Jones, 1990; Cole et al., 1993; Li et al., 1994), the cellular pathobiology underlying cerebellar dysfunction remains an under-investigated area.

The cerebellum has a well-established role in the coordination of movements (Houk and Miller, 2001; Morton and Bastian, 2004; Koziol et al., 2014) but also plays a role in higher order functions, including emotion, language, and cognition (Gordon, 1996; de Smet et al., 2007, 2013; Turner et al., 2007). Morphologically, the cerebellum is a tri-laminar structure comprised of a superficial molecular layer mainly containing basket and stellate interneurons, a middle cell layer consisting of a monolayer of Purkinje cells (PCs) and a deep granular cell layer containing small excitatory granule cells (e.g., brush cells) and inhibitory (Golgi and Lugaro) interneurons (Roostaei et al., 2014). Cerebellar gamma-aminobutyric acid (GABA) inhibitory PCs provide the major cerebellar output to the deep cerebellar and vestibular nuclei (Herndon, 1963; Delgado-Garcia, 2001; Houk and Miller, 2001). Cerebellar excitatory inputs (Wadiche and Jahr, 2001; Bagnall and du Lac, 2006; Arenz et al., 2009; Kurtaj et al., 2013; Roostaei et al., 2014) that arise from the brainstem/spinal cord and inferior olivary nuclei course within the mossy fiber-granule cell parallel and climbing fibers, respectively (Hoxha et al., 2018). PCs contain the calcium-binding proteins (CBPs) calbindin D-28k (Calb) and parvalbumin (Parv), while calretinin (Calr) is found exclusively in cells within the cerebellar granule cell layer (Bastianelli, 2003). CBPs maintain intracellular calcium homeostasis, which play a key role in synaptic function (Iacopino and Christakos, 1990; Airaksinen et al., 1997; Caillard et al., 2000; Bastianelli, 2003; Gattoni and Bernocchi, 2019). Disruption of cellular homeostasis, due to an increase of free cytoplasmic calcium, induces neuronal apoptosis (Orrenius et al., 2003). Although it was hypothesized that calcium dysregulation plays a major role in the cellular pathogenesis of AD (Khachaturian, 1994), less is known about its actions in DS.

Reduction in Calb and Parv containing neurons has been reported in the frontal and temporal cortex of adults with DS (Kobayashi et al., 1990). Interestingly, knockout of Calb and Parv in PCs impairs motor coordination and sensory processing (Airaksinen et al., 1997; Barski et al., 2003) with the most severe deficits occurring when both proteins are deleted in rodents (Farré-Castany et al., 2007). Furthermore, Calr null mice display impaired granule cells and PC function (Schiffmann et al., 1999), while the induction of Calr restores normal cerebellar function (Bearzatto et al., 2006). These data suggest that disturbances in CBPs within the neurons of cerebellar cortex contribute to motor and cognitive impairment in DS. However, the effects of trisomy upon CBP containing neurons in the cerebellum of individuals with DS remain unclear.

In addition to CBPs, PCs also contain the cognate receptors TrkA and p75^NTR^ for the neuronal survival protein, nerve growth factor (NGF) (Mufson et al., 1991; Savaskan et al., 2000). Although these receptors are constitutively expressed during early cerebellar development through adulthood (Muragaki et al., 1995; Roux and Barker, 2002; Quartu et al., 2003a,b; Florez-McClure et al., 2004; Schor, 2005; Lotta et al., 2014), their role in PC pathology in DS is poorly defined. Numerous studies indicate that NGF receptors play a key role in cholinergic basal forebrain neuron dysfunction in AD and DS (Sendera et al., 2000; Mufson et al., 2019). However, the effect of trisomy upon TrkA and p75^NTR^ and their relationship with CBPs in PCs in DS remains to be investigated. Therefore, the present study examined CBPs and NGF receptors in the cerebellar cortex in DS and AD compared to healthy non-dementia subjects (HC) using quantitative immunohistochemistry, densitometry, and morphometry.

## Materials and Methods

### Subjects

Cerebellar cortex from a total of 30 adults (44–98 years of age) who died with an ante-mortem clinical diagnosis of AD, DS, or HC was obtained from the Rush University Department of Pathology, Chicago, IL (8 HC, 10 AD, and 5 DS cases), University of California at Irvine Alzheimer's Disease Research Center (UCI ADRC; 6 DS cases), and the Barrow Neurological Institute at St. Joseph's Hospital and Medical Center, Phoenix, AZ, USA (BNI; 1 DS case). Of the 12 DS cases, 9 had dementia (DSD+), and 3 did not (DSD+) and three were non-demented (DSD–). Tissue collection and handling conformed to the guidelines of each respective Institutional Review Board (IRB) protocol. DS diagnosis was confirmed by the presence of an extra copy of HSA21 using fluorescence *in situ* hybridization and/or chromosome karyotyping.

Dementia status was determined as previously reported (Perez et al., 2019). Briefly, the clinical status of the UCI ADRC DS participants was determined in accordance with International Classification of Diseases and Related Health Problems-Tenth Revision (ICD-10) and Dementia Questionnaire for Mentally Retarded Persons (DMR-IV-TR) criteria (Sheehan et al., 2015). All UCI ADRC and the BNI DS cases were participants in longitudinal research protocols prior to death. Assessments included physical and neurological exams and a history obtained from both the participant and a reliable caregiver. Standardized direct and indirect cognitive and behavioral assessments were also completed. The diagnosis of dementia required deficits in two or more areas of cognitive functioning and progressive worsening of cognitive performance compared to the baseline performance of an individual. Cases with cognitive decline due to confounding factors that may mimic dementia (e.g., depression, sensory deficits, and hypothyroidism) were eliminated. Premorbid-intelligence quotient (IQ) was also determined in all the UCI DS cases. Determination of clinical status of the Rush cases was performed by a neurologist trained in gerontology together with discussions with a caregiver. Human Research Committees of Rush University Medical Center, University of California at Irvine, and Barrow Neurological Institute approved this study. Supplementary Table 1 details the clinical, demographic, and neuropathological features and tissue source of the cases used in this study.

### Neuropathological Evaluation

Since virtually all of the tissue utilized in this study was obtained from archival cases collected prior to the establishment of the National Alzheimer's Coordinating Center, National Institute on Aging (NIA)-Reagan criteria (Newell et al., 1999), Consortium to Establish a Registry for Alzheimer's Disease (CERAD) (Mirra et al., 1991), and Thal amyloid staging (Thal et al., 2002) were not available. Neuropathological diagnosis was based on Braak staging of NFTs (Braak and Braak, 1991). None of the cases examined were treated with acetylcholinesterase inhibitors.

### Tissue Processing

The cerebellum was immersion fixed in either 4% paraformaldehyde or 10% formalin for 3–10 days, cut on a sliding freezing microtome at 40 μm thickness, and stored in cryoprotectant (40% phosphate buffer pH7.4, 30% glycerol, and 30% ethylene glycol) at −20°C prior to processing as previously reported (Perez et al., 2019). All free-floating sections were mounted on positive charged slides and air-dried overnight prior to the histochemical [hematoxylin and eosin (H&E) and cresyl violet] and immunohistochemical procedures.

### Immunohistochemistry

Antibody characteristics, dilution, and the commercial company from which each was purchased are shown in Table 1. Before immunostaining, cerebellar sections were pretreated for antigen retrieval with boiling citric acid (pH 6) for 10 min for Calb, Parv, Calr, SMI-32, SMI-34 and 15 min for TrkA and p75^NTR^. Antigen retrieval for the 6E10, Aβ_40_, and Aβ_42_ antibodies consisted of placing sections in 88% formic acid at room temperature (RT) for 10 min. Sections were then washed in Tris-buffered saline (TBS, pH 7.4) followed by incubation in 0.1 M sodium metaperiodate (Sigma-Aldrich, St. Louis, MO, USA) to inactivate endogenous peroxidases, permeabilized in TBS containing 0.25% Triton X-100 (Thermo Fisher Scientific, Waltham, MA, USA) and blocked in TBS/0.25% Triton containing 3% goat serum for 1 h. Sections were incubated with primary antibodies overnight at RT in TBS containing 0.25% Triton X-100 and 1% goat serum. The next day, after three washes with TBS/1% goat serum, sections were incubated with affinity-purified goat anti-mouse or goat anti-rabbit biotinylated secondary antibodies (Vector Laboratories, Burlingame, CA, USA) for 1 h at RT. After washes in TBS, sections were incubated in Vectastain Elite ABC kit (Vector Laboratories) for 1 h at RT and developed in acetate-imidazole buffer containing the chromogen 0.05% 3,3′-diaminobenzidine tetrahydrochloride (DAB; Sigma-Aldrich). Immunoreactivity for TrkA and p75^NTR^ was enhanced using a solution consisting of DAB and nickel sulfate (0.5–1.0%). The reaction was terminated in acetate-imidazole buffer (pH 7.4), and sections were dehydrated in graded alcohols, cleared in xylenes, and cover-slipped with DPX mounting medium (Electron Microscopy Sciences, Hatfield, PA, USA). Cytochemical controls consisted of the omission of primary antibodies, which resulted in no detectable immunoreactivity (see Supplementary Figures 1A–D). In addition, a series of control experiments were performed to demonstrate that the TrkA antibody used in this study does not immunostain neurons containing TrkB. First, as a positive control for both TrkA and the p75^NTR^ immunolabeling, we stained sections containing the cholinergic neurons within the nucleus basalis of Meynert (nbM), which display both of these proteins (Mufson et al., 1989; Perez et al., 2012), obtained from a female 93-year-old HC and a female 94-year-old AD case, respectively. Supplementary Figures 1E,F show positive cellular nbM reactivity for each antibody. Secondly, sections containing neurons located within the oculomotor nucleus (cranial nerve III) and the substantia nigra (SN) of a male 51-year-old HC case were immunostained using the current TrkA antibody. Although oculomotor neurons also displayed TrkA immunoreactivity, SN neurons, which contain TrkB (Jin, 2020), were TrkA (Sobreviela et al., 1994) immunonegative (Supplementary Figures 1G,H). Some sections were counterstained with Gill's hematoxylin or cresyl violet for laminar identification.

**Table 1:** Antibody characteristics.

**Antigen**	**Primary antibody**	**Dilution IH (IF)**	**Company: Catalog#**	**Secondary antibody** **Company: Catalog#**
Tau (AT8)	Mouse monoclonal to phosphorylated-tau (Ser202/Thr205)	1:1,000	Invitrogen: MN1020	Biotinylated goat anti-mouse IgG Vector Laboratories: BA9200
APP/Aβ	Mouse monoclonal to residues 1–16 of N-terminus human Aβ (6E10)	1:1,000	Biolegend: 803002	
Aβ_40_	Rabbit polyclonal to 7 aa peptide sequence from C-terminus of human Aβ1–40	1:1,000	Millipore: AB5074P	Biotinylated goat anti-rabbit IgG Vector Laboratories: BA1000 ^*^Cy2-Donkey anti-rabbit IgG Jackson ImmunoResearch Laboratories: 711225152
Aβ_42_	Rabbit polyclonal to 6 aa peptide sequence from C-terminus of human Aβ1–42	1:1,000	Millipore: AB5078P	
^*^Calbindin D-28k	Rabbit polyclonal to 28 kD calcium-binding protein	1:15,000 (1:1,000)	Swant: CB38	
Parvalbumin	Rabbit polyclonal made from purified parvalbumin	1:1,000	Novus: NB120-11427	
Calretinin	Rabbit polyclonal to 99 aa epitope from the internal region of rat calretinin	1:1,000	Millipore: ABN2191	
TrkA	Rabbit polyclonal to extracellular domain of rat TrkA receptor	1:500	Millipore: 06-574	
p75^NTR^	Mouse monoclonal to aa 1–160 from A875 melanoma cells; Clone NGFR Ab-1	1:500	NeoMarkers/Thermo Scientific MS-394-P1	Biotinylated goat anti-mouse IgG Vector Laboratories: BA9200 ^*^Cy3 Donkey anti-mouse IgG Jackson ImmunoResearch Laboratories: 715165151
^*^SMI-32	Mouse monoclonal to anti-Neurofilament H, non-phosphorylated	1:2,000 (1:500)	Biolegend: 801701	
SMI-34	Mouse monoclonal to anti-Neurofilament H, phosphorylated	1:1,000	Biolegend: 835503	
^*^Parvalbumin	Mouse monoclonal produced with purified parvalbumin	(1:50)	Swant: PV235	^*^Cy3 Donkey anti-mouse IgG Jackson ImmunoResearch Laboratories: 715165151

* *Fluorescent primary and secondary antibodies; IH, immunohistochemistry; IF, immunofluorescence*.

### Immunofluorescence

To evaluate the relationship between Calb, Parv, and SMI-32, cerebellar sections were mounted onto positive charged slides, pretreated with boiling citric acid (pH6) for 10 min for antigen retrieval, washed with TBS, blocked in a TBS/0.5%Triton solution containing 3% donkey serum, and dual-labeled with rabbit anti-Calb and mouse anti-Parv or mouse anti-SMI-32. Sections were incubated overnight for Calb at RT washed with TBS/1% donkey serum and incubated in Cy2-conjugated donkey anti-rabbit immunoglobulin G (IgG) secondary antibody (1:200, Jackson ImmunoResearch, West Grove, PA, USA) for 1 h. After several washes with TBS/1% donkey serum, sections were incubated overnight using either anti-Parv or anti-SMI-32 at RT and then placed in Cy3-conjugated donkey anti-mouse IgG secondary antibody for 1 h (1:200, Jackson ImmunoResearch). Following development, sections were washed in TBS, dehydrated in graded alcohols, cleared in xylenes, and cover-slipped with DPX mounting medium. Fluorescence was visualized with the aid of a Revolve Fluorescent Microscope (Echo Laboratories, San Diego, CA, USA) with excitation filters at wavelengths 489 and 555 nm for Cy2 and Cy3, respectively.

### Quantitative Morphometric and Densitometric Measurements

Purkinje cells were quantified in H&E and cresyl violet stained sections in 10 randomly selected fields in a section using a 20× objective with an area of 0.20 mm^2^ per field and presented as mean counts. Thickness of the cerebellar granular cell layer (GL) and molecular layer (ML) was quantified in 10 randomly chosen fields from cresyl violet stained sections using the same parameters as described above. Calb-, Parv-, SMI-32-, TrkA-, and p75^NTR^-ir PC, ML Parv-ir interneuron, and Calr-ir Golgi, Lugaro, and unipolar brush cell counts in the GL were performed in two sections, 10 fields per section at 20× magnification, and calculated as mean per section. Non-phosphorylated SMI-32- and phosphorylated SMI-34-immunolabeled fusiform PC axonal swellings (i.e., torpedoes) in the GL, ML, and PC layers were quantified in one entire section per case at 10× magnification and reported as mean number of torpedoes per total cerebellar area (cm^2^). Optical density (OD) measurements of Calb, Parv, SMI-32, p75^NTR^, and TrkA PC neurons were measured in 10 fields per section within two sections at 40× magnification covering an area of 0.40 mm^2^/per field and calculated as means per section. Additional OD measurements of Calb- and p75^NTR^-ir ML dendritic arborization were quantified in 10 fields per section at 40× magnification as described above. Background measurements were averaged and subtracted from the mean OD values for each immunostained section. Quantitation and photography were performed with the aid of a Nikon Eclipse microscope coupled with NIS-Elements imaging software (Nikon, Japan).

APP/Aβ (6E10), and Aβ_42_-ir plaque load were evaluated in two sections within ten randomly selected fields per section at 20× magnification covering an area of 0.20 mm^2^/per field within the ML. Plaque load was calculated as percent area per cerebellar field and presented as mean number per section.

### Statistical Analysis

Analysis of cell counts, OD measurements, and demographic differences between HC, DS, and AD cases were evaluated using Chi-squared (comparing gender), non-parametric Mann–Whitney rank test, and Kruskal–Wallis ANOVA on ranks test, followed by Dunn's *post-hoc* test for multiple comparisons. Correlations between variables and demographics were performed using a Spearman's test. Data significance was set at *p* < 0.05 (two-tailed). A sub-analysis was performed excluding the DSD– subjects from the DS group. Group differences for each marker were adjusted for age and gender using an analysis of covariance (ANCOVA) with and without DSD– cases after log-transforming variables that did not meet the assumption of normality. For both ANCOVAs, false discovery rate (FDR) was used to correct for multiple comparisons and α was set at < 0.01.

## Results

### Case Demographics

Average age for each group was 70.90 (±12.60) for HC, 81.70 (±8.00) for AD, and 51 (±6.4) years for DS cases. Statistical analysis revealed a significantly lower age for the DS (*p* < 0.05) compared to both the HC and AD subjects (Table 2). There was no significant difference in gender frequency between groups (Chi-squared, *p* = 0.9). Average brain weight was significantly lower for DS (954.50 ± 121.90 g) than the HC (1259.40 ± 70.73 g) (Kruskal–Wallis, *p* = 0.002) but not compared to AD (1108.50 ± 177.00 g) (Table 2). A significantly higher post-mortem interval was found for the HC (PMI, 13.88 ± 5.40 h) compared to AD, (5.20 ± 1.19 h) (Kruskal–Wallis, *p* < 0.05) but not DS (7.48 ± 5.58 h) (Table 2). Braak NFT stages were significantly higher in DS and AD compared to HC cases (Kruskal–Wallis, *p* ≤ 0.001) (Table 2). DSD+ was Braak VI compared to Braak V for the DSD– cases. For AD, 77% were Braak VI and 23% Braak V. Within the HC group 50% had a Braak score of I–III, while the others were stage IV–V. These latter cases may have brain reserve allowing for the clinical diagnosis of no dementia (Mufson et al., 2016).

**Table 2:** Case demographics.

	**HC (*n* = 8)**	DS (*n* = 12)^*^	**AD (*n* = 10)**	***p*-value**	**Group-wise comparisons (*p*-value)**
Age (years)	70.88 ± 12.55 [51–85]^a^	51.00 ± 6.41 [44–60]	81.70 ± 7.97 [71–98]	<0.001^b^	DS < HC (0.011) DS < AD (<0.001)
Male/Female (n)	3/5	4/8	3/7	0.9^c^	—
PMI (h)	13.88 ± 5.41 [3–20]^a^	7.48 ± 5.58 [2.2–20]	5.20 ± 1.18 [3.5–6]	0.029^b^	AD < HC (0.037)
Brain weight (g)^**^	1259.40 ± 70.73 [1180–1400]^a^	954.40 ± 121.92 [700–1090]	1108.50 ± 177.00 [925–1430]	0.003^b^	DS < HC (0.002)
Braak scores (n)^***^	I [1], II [2], III [1], IV [3], V [1]	V [3], VI [9]	V [2], VI [7]	≤0.001^b^	HC < AD, DS (≤0.001)

*Dementia DS n = 9; non-dementia DS n = 3*.

** *1 dementia DS subject and 1 non-dementia DS subject do not have brain weight data*.

*** *1 AD subject does not have a Braak score*.

a *Mean ± SD [range]*.

b *Kruskal–Wallis test*.

c *Chi-squared*.

### Cerebellar Amyloid and Tau Pathology

**Figure 1:**
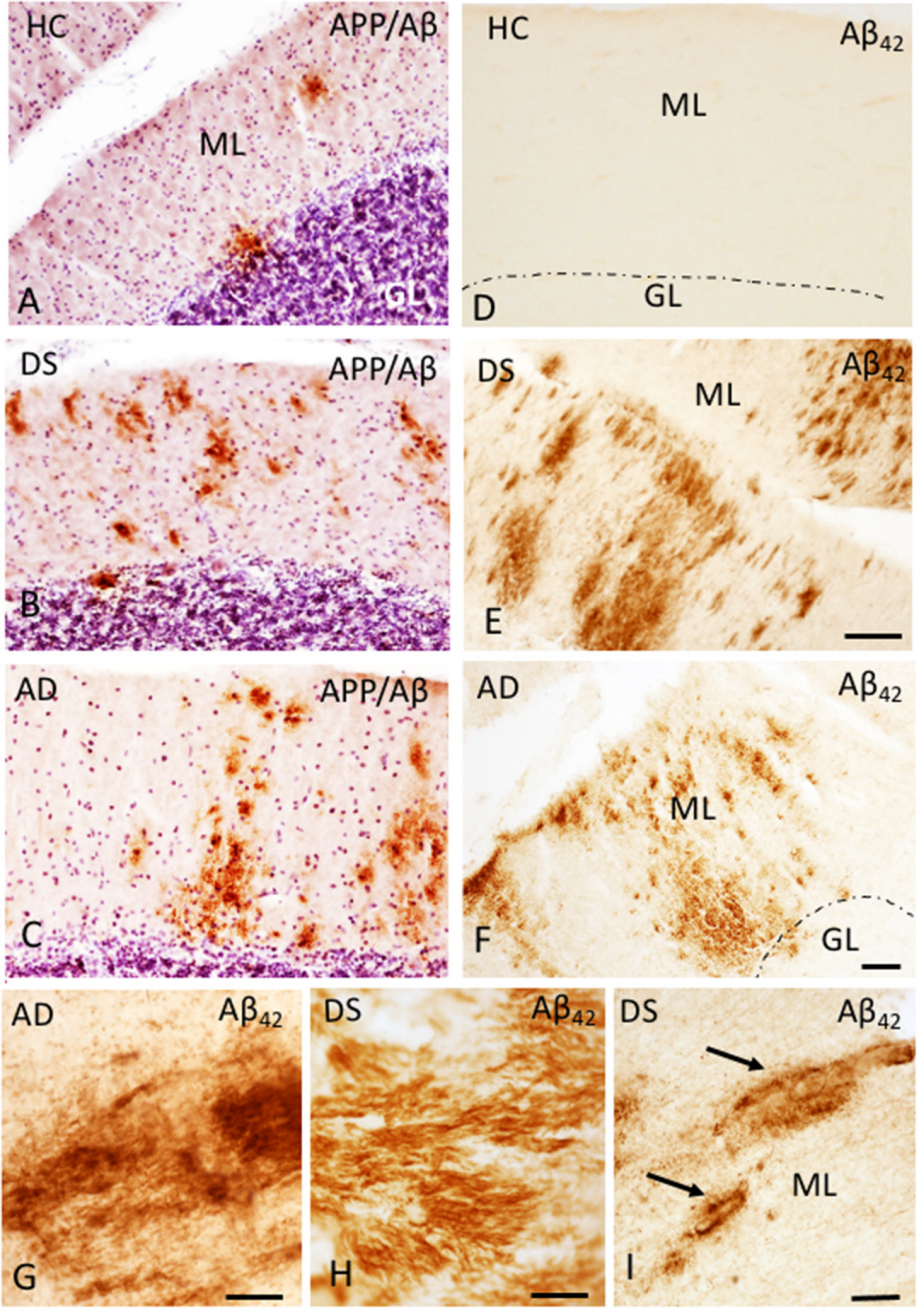
Photomicrographs showing a few scattered APP/Aβ- **(A)** and the absence of Aβ_42_-ir **(D)** plaques in the ML of the cerebellar cortex of a 66-year-old female HC **(A,D)** compared to numerous APP/Aβ- **(B,C)** and Aβ_42_-ir **(E,F)** plaques in a 46-year-old male with DSD+ **(B,E)** and a 98-year-old female with AD **(C,F)** case, respectively. High-power photomicrographs of Aβ_42_-ir filament-like bundles within diffuse plaques in a 98-year-old female with AD **(G)** and 47-year-old female with DSD– **(H)**, and Aβ_42_-ir blood vessels (arrows) in a 46-year-old male with DSD+ **(I)**. **(A–C)** were counterstained with Gill's hematoxylin to aid in the visualization of cerebellar laminae. HC, healthy control; DS, Down syndrome; AD, Alzheimer's disease; ML, molecular layer; GL, granular cell layer. Scale bars: **(E)** = 100 μm applies to **(A,B,D)**; **F** = 100 μm applies to **(C)**; **G,H** = 10 μm; **(I)** = 50 μm.

The presence of Aβ plaques and NFTs in the cerebellar cortex was determined using antibodies against APP/Aβ (6E10), Aβ_40_, Aβ_42_, and AT8, an antibody that marks tau phosphorylation. Although APP/Aβ-ir deposits were found in each HC, DS, and AD case, only 12.5% of HC and 70% of AD cases displayed Aβ_42_-ir plaques. On the other hand, Aβ_42_-ir plaques were observed in both DSD+ and DSD– cases. Qualitative evaluation revealed diffuse aggregates of APP/Aβ-ir plaques scattered within the ML in both DS and AD (Figures 1B,C) compared to small rounded APP/Aβ-ir deposits in the GL and PC layers in both groups, while very few plaques were found in HC cases (Figure 1A). Aβ_42_-ir plaques were observed mainly in the ML (Figures 1E,F) in both DS and AD but were not observed in HC (Figure 1D). Diffuse Aβ_42_- and APP/Aβ-ir plaques within the ML displayed amyloid positive filaments (Figures 1G,H). In addition, APP/Aβ and Aβ_42_-ir leptomeningeal arteries, arterioles, and/or capillaries (Figure 1I) were observed in 75% of DS, 30% of AD, and 12.5% of HC cases. Aβ_40_ immunoreactivity was observed in leptomeningeal arteries in 66% of DS, 30% of AD, and 12.5% of HC cases. Interestingly, PCs were Aβ_42_ and APP/Aβ immunonegative and neither Aβ_40_ plaques nor AT8-ir profiles were observed in the cerebellar cortex across groups.

### Quantitation of Amyloid Plaque Load

**Figure 2:**
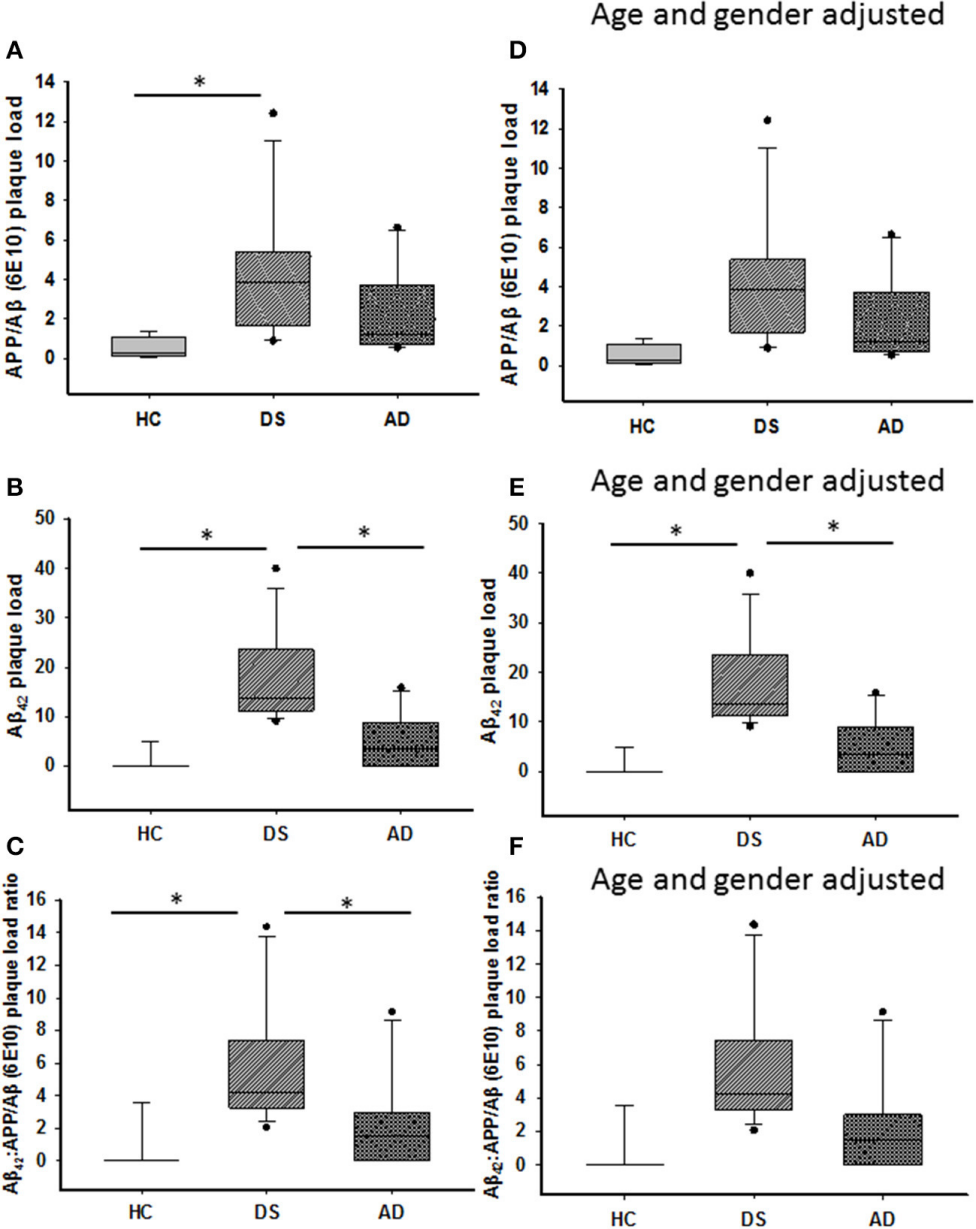
Box plots showing significantly higher APP/Aβ- **(A)** and Aβ_42_-ir **(B)** plaque loads, as well as Aβ_42_:APP/Aβ **(C)** plaque load ratio in the ML of DS compared to HC cases (Kruskal–Wallis, *p* < 0.01), while Aβ_42_-ir plaque load (**B**; Kruskal–Wallis, *p* = 0.01) and Aβ_42_:APP/Aβ plaque load ratio (**C**; Kruskal–Wallis, *p* = 0.04) were significantly greater in DS compared to AD. Adjusting for age and gender revealed a greater ML Aβ_42_ plaque load in DS compared to HC and AD (**E**; ANCOVA, *p* = 0.001), with no difference in APP/Aβ **(D)** plaque load and Aβ_42_:APP/Aβ **(F)** ratio between groups (ANCOVA, *p* > 0.01). ANCOVA, analysis of covariance. * denotes significance between groups.

Amyloid plaque load was measured within the ML using APP/Aβ (6E10) and Aβ_42_ antibodies. Aβ_42_-ir plaque load was significantly higher than APP/Aβ in DS with or without dementia (Mann–Whitney, *p* ≤ 0.001), while no differences were detected between Aβ_42_ and APP/Aβ load in AD (data not shown). HCs displayed a higher APP/Aβ than Aβ_42_ load (Mann–Whitney, *p* < 0.01, data not shown). APP/Aβ- (Figure 2A) and Aβ_42_-ir (Figure 2B) plaque loads were significantly increased in DS compared to HC cases (Kruskal–Wallis, *p* < 0.01), while DS Aβ_42_-ir plaque load was greater than AD (Kruskal–Wallis, *p* = 0.01). Similar results were observed when DSD– cases were removed from the statistical analysis for both antibodies. Adjusting for age and gender revealed a significantly greater Aβ_42_ plaque load in DS compared to HC (ANCOVA, *p* = 0.001) and AD (ANCOVA, *p* = 0.001) (Figure 2C) but no difference in APP/Aβ plaque load (ANCOVA, *p* > 0.01) (Figure 2D) between groups. A sub-analysis removing DSD– cases showed a significantly greater Aβ_42_ (ANCOVA, *p* < 0.001) and APP/Aβ plaque (ANCOVA, *p* = 0.03) load in DSD+ compared to HC. Furthermore, the ratio between Aβ_42_ and APP/Aβ-ir plaque load was greater in DS compared to HC (Kruskal–Wallis, *p* < 0.001) (Figure 2E) and AD (Kruskal–Wallis, *p* < 0.04) (Figure 2E). Eliminating DSD– cases from the analysis resulted in a significantly higher Aβ_42_:APP/Aβ-ir plaque load ratio in DSD+ than HC cases (Kruskal–Wallis, *p* < 0.001) but not in AD (Kruskal–Wallis, *p* < 0.001). Adjusting for age and gender, with and without DSD–, revealed no differences in Aβ_42_:APP/Aβ-ir plaque load ratio between groups (ANCOVA, *p* > 0.01) (Figure 2F).

### Quantitation of H and E and Cresyl Violet Stained PC Cells

**Figure 3:**
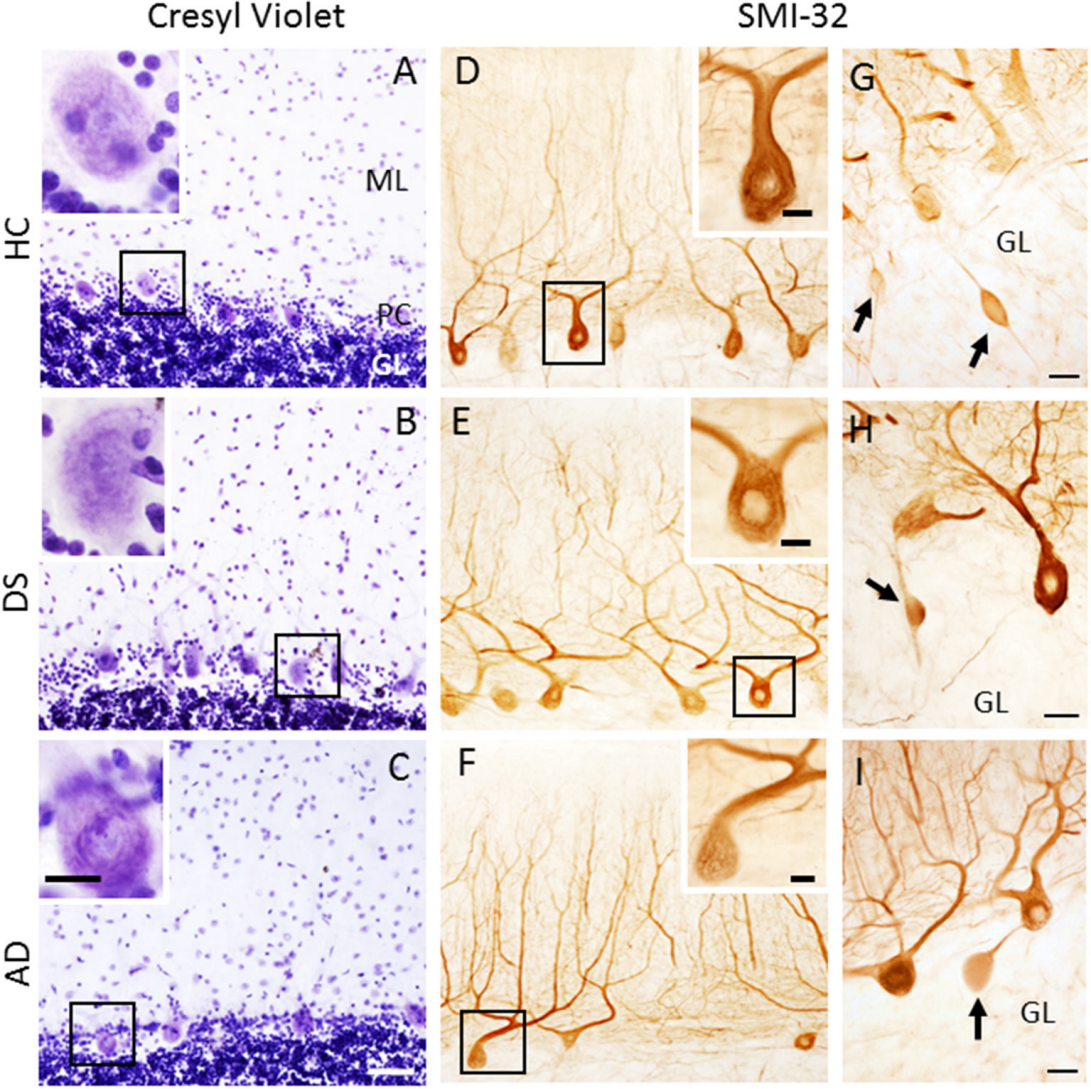
Low-power photomicrographs of cresyl violet staining of the cerebellar cortex showing the ML, PC layer, and GL in 63-year-old male HC **(A)**, 46-year-old male with DSD+ **(B)**, and 79-year-old female with AD **(C)** cases. Insets in the upper left corners show high-power images of PC perikaryon outlined in the black boxes in **(A–C)**. Note the absence of the PC dendritic arbor **(A–C)**. Photomicrographs of SMI-32-ir non-phosphorylated high-molecular-weight neurofilaments in PC dendritic arbors and axons in a female 69-year-old HC **(D)**, male 46-year-old DSD+ **(E)** and a male 85-year-old AD **(F)** case. Insets in **(D–F)** show high-power images of boxed SMI-32-ir PCs and proximal dendrites. **(G–I)** Swollen SMI-32-ir proximal PC axons or torpedoes (arrows) in the GL of a male 51-year-old HC **(G)**, 60-year-old female with DS **(H)** and 98-year-old female with AD **(I)** case. Scale bars: **(C)** = 50 μm applies to **(A,B,D–F)**; inset in **(C)** = 15 μm, insets in **(A,B,D–F)** = 10 μm; **(G–I)** = 25 μm.

**Figure 4:**
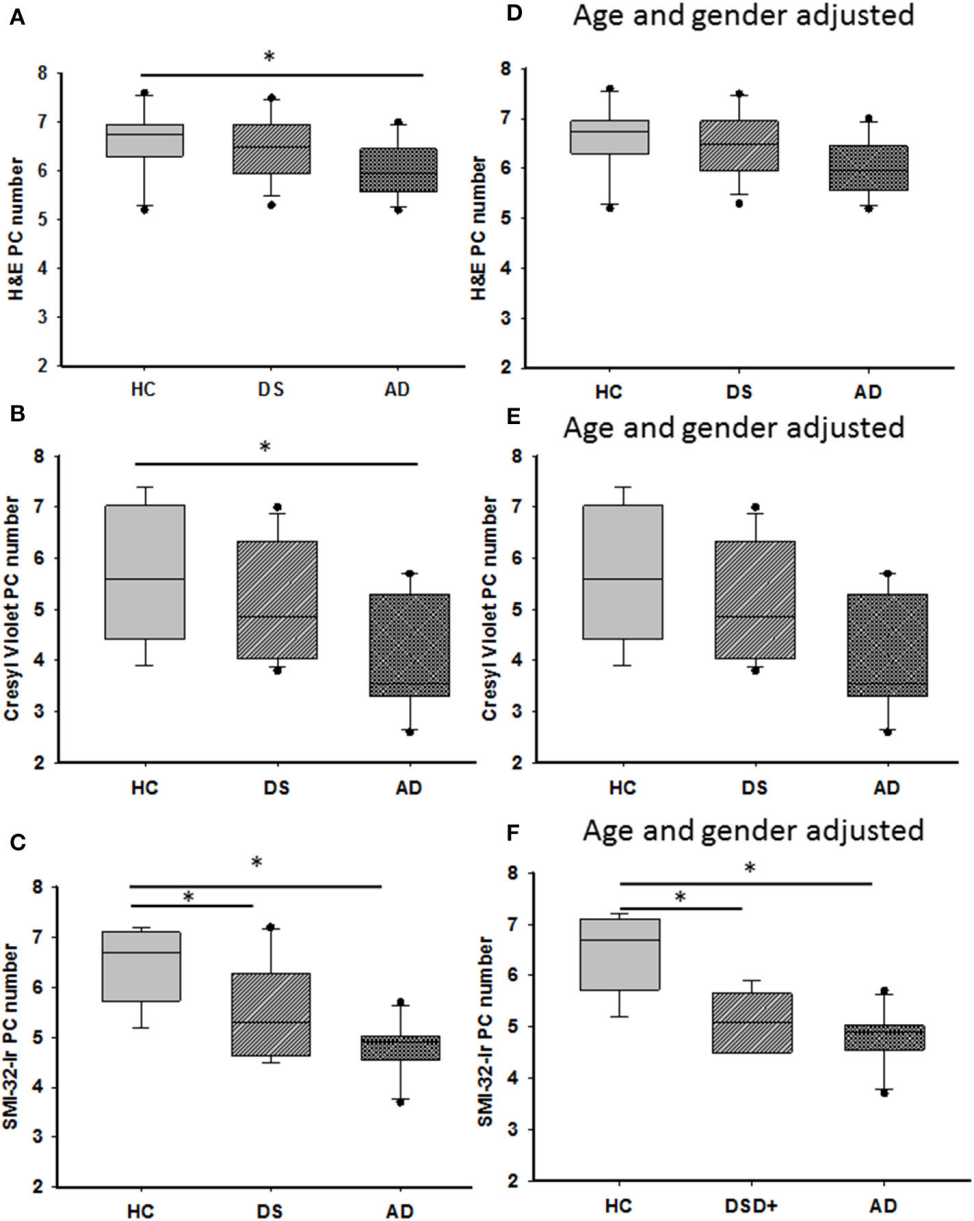
Representative box plots showing a significant reduction in H&E **(A)** and cresyl violet **(B)** stained and SMI-32 immunoreactive **(C)** PCs in AD compared to HC cases (Kruskal–Wallis, *p* < 0.05), as well a significant reduction in the number of SMI-32-ir PCs in DS compared to HC (**C**; Kruskal–Wallis, *p* = 0.001). Adjusting for age and gender revealed that the number of H&E **(D)** and cresyl violet **(E)** stained PCs remained unchanged between groups as did counts for SMI-32 positive PCs (**F**; ANCOVA, *p* < 0.001). H&E, hematoxylin and eosin. * denotes significance between groups.

H&E and cresyl violet stained sections were used to count PCs. GL and ML thickness were evaluated using cresyl violet due to greater laminar differentiation. In all groups, H&E and cresyl violet stained PCs but not dendrites (Figures 3A–C). Surprisingly, quantitation revealed fewer cresyl violet compared to H&E positive PCs in DS and AD compared to HC (Mann-Whitney, *p* < 0.01, data not shown). There was also a significant reduction in number of H&E and cresyl violet stained PCs in AD compared to HC cases (Kruskal–Wallis, *p* < 0.05) (Figures 4A,B), but not DS. Furthermore, there were no significant differences in GL and ML laminar thickness between groups (Kruskal–Wallis, *p* > 0.05, data not shown). A sub-analysis removing DSD– cases showed similar findings. Adjusting for age and gender revealed no significant differences in the number of H&E and cresyl violet stained PCs (Figures 4D,E) nor GL and ML thickness between groups, even when DSD– cases were removed from the evaluation. In addition, no significant differences were found in the number of cresyl violet compared to H&E-stained PCs between DS groups (Kruskal–Wallis, *p* > 0.05) (Supplementary Figure 2A), even after adjusting for age and gender (ANCOVA, *p* > 0.01; Supplementary Figure 2B).

### SMI-32 and SMI-34 Neurofilament PC Counts

**Figure 5:**
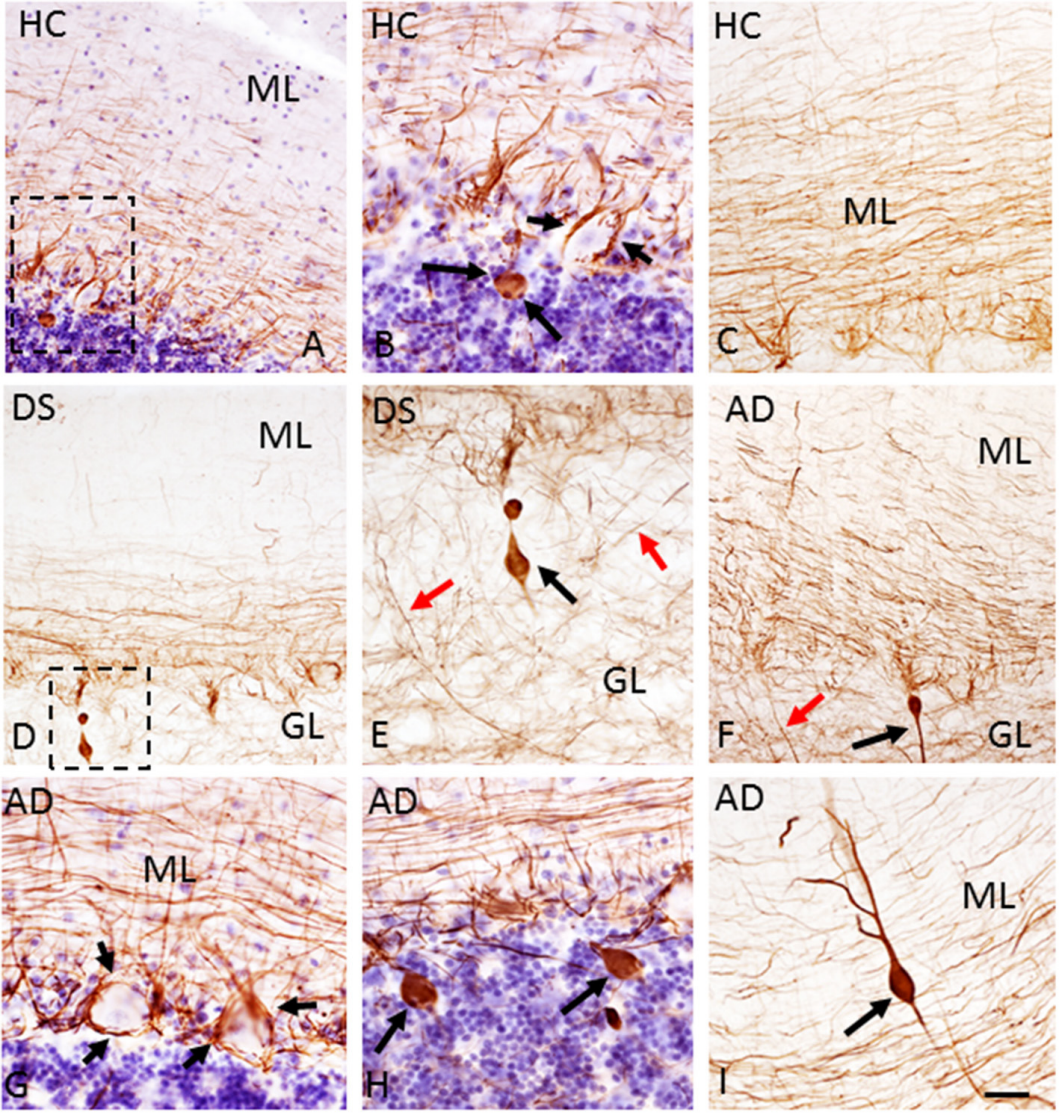
Photomicrographs showing SMI-34-immunoreactivity in parallel fibers, basket cell axons around PCs (**B,G**; small arrows), PC axons (**E,F**; red arrows) and torpedoes (**A,B,D–F,H,I**; larger black arrows) in a 51-year-old male HC **(A,B)**, **(C)** 85-year-old male HC, a 47-year-old female with DSD– **(D,E)**, a 79-year-old female **(G,H)**, and 88-year-old male with AD **(F,I)** case. **(B,E)** Show high-power images of the cells outlined by the dotted boxes in **(A,D)**, respectively. Note the lack of SMI-34 immunostaining in PCs across groups **(A–H)** and an increase in SMI-34-ir parallel fibers in the ML of a 85-year-old male HC **(C)** compared to a 51-year-old male HC **(A)** and a 47-year-old female with DS **(D)**. Note a rare dendritic torpedo in the ML of a 78-year-old female with AD **(I)**. Tissue shown in **(A,B,G,H)** were counterstained with Gill's hematoxylin. Scale bar = 50 μm in **(A,C,D,F)** and 20 μm in **(B,E,G–I)**.

The number of PCs was also examined using antibodies against SMI-32, a somato-dendritic neuronal marker that preferentially labels non-phosphorylated neurofilaments in soma and dendrites. SMI-34 recognizes phosphorylated intermediate neurofilaments of high-molecular-weight. SMI-32 immunostaining was observed in PC soma, dendrites, and axons (Figures 3D–F) in all groups. SMI-34 immunostained basket cell (Figures 5B,G) axons, PC axons (Figures 5E,F), and parallel fibers across cohorts (Figures 5A,C,D,F–I). Although PC soma were SMI-34 immunonegative (Figures 5B,G), we observed an increase in SMI-34 immunopositive parallel fibers within the superficial portion of the ML in the oldest cases independent of group (Figures 5A,C,D).

Quantitative analysis revealed a significant decrease in the number of SMI-32-ir PCs in AD compared to HC (Kruskal–Wallis, *p* = 0.001) (Figure 4C) but not in DS. A sub-analysis removing DSD– cases found a significant reduction in SMI-32-ir PCs in DSD+ compared to HC cases (Kruskal–Wallis, *p* = 0.02). SMI-32-ir PC ODs were significantly higher in DS compared to HC (*p* = 0.029) but not AD subjects. This significant difference was lost when the DSD– cases were eliminated from the analysis (*p* = 0.09). Adjusting for age and gender, HC subjects displayed a significantly higher number of positive SMI-32 PCs than AD or DS with or without dementia (ANCOVA, *p* < 0.001) (Figure 4F), with no difference in SMI-32-ir PC OD intensity among groups (ANCOVA, *p* > 0.01) with or without dementia DS cases. Furthermore, the ratio of the number of SMI-32-ir PCs to H&E PC numbers in AD and DS with (Kruskal–Wallis, *p* = 0.03) or without dementia (Kruskal–Wallis, *p* = 0.04) was lower compared to HC cases (Supplementary Figure 2C). Adjusting for age and gender yielded a similar result only when DSD– cases remained in the analysis (ANCOVA, *p* = 0.01, Supplementary Figure 2D).

**Figure 6:**
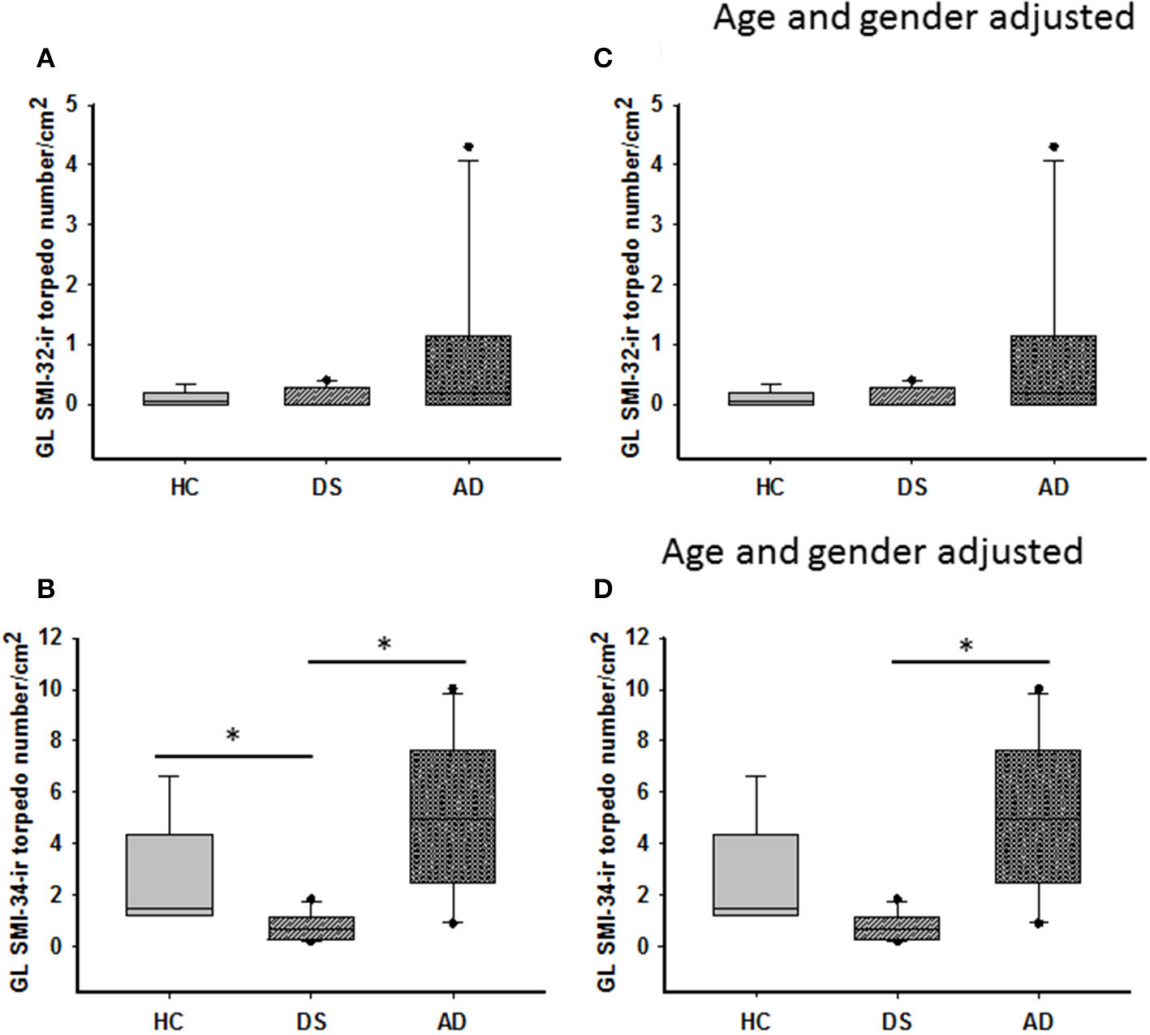
Box plots showing no difference in the number of GL SMI-32 stained axonal torpedoes between groups **(A)** even after adjusting for age and gender **(C)**. A significant decrease in GL SMI-34-ir torpedo numbers was observed in DS compared to HC and AD cases (**B**; Kruskal–Wallis, *p* < 0.001). When adjusted for age and gender, GL SMI-34-ir axonal torpedo counts were significantly reduced in DS compared to AD, but not HC (**D**; ANCOVA, *p* < 0.001). * denotes significance between groups.

SMI-32 and SMI-34 immunostaining also revealed swellings mainly in proximal PC axons termed torpedoes/spheroids (Bouman, 1918) (Figures 3G–I, 5E,F). While SMI-32-ir PC torpedoes were found primarily in the GL in 60% of AD, 33% of DS, and 25% of HC cases, SMI-34-ir torpedoes were observed in all three cerebellar layers in each group. Although SMI-34-ir torpedoes were less abundant in both ML and PC layers compared to the GL, a few were seen in PC dendrites (Figure 5I). Counts of SMI-34-ir torpedoes in the GL revealed significantly higher numbers in both AD and HC cases compared to DS (Kruskal–Wallis, *p* < 0.001) (Figure 6B), even when DSD– cases were removed from the analysis (Kruskal–Wallis, *p* < 0.001), while SMI-32 torpedo numbers were similar between groups (Kruskal–Wallis, *p* > 0.05) (Figure 6A). Controlling for age and gender yielded similar results for SMI-32 (Figure 6C), while the number of SMI-34-ir torpedoes were significantly higher in AD compared to DS independent of dementia, but not HC (ANCOVA, *p* = 0.01) cases (Figure 6D). Furthermore, the number of SMI-34-ir torpedoes were significantly higher than the number of SMI-32 torpedoes in all three groups (Mann–Whitney, HC *p* < 0.001, DS *p* = 0.024, and AD *p* = 0.004, data not shown).

### Calbindin, Parvalbumin, and Calretinin Cell Counts

**Figure 7:**
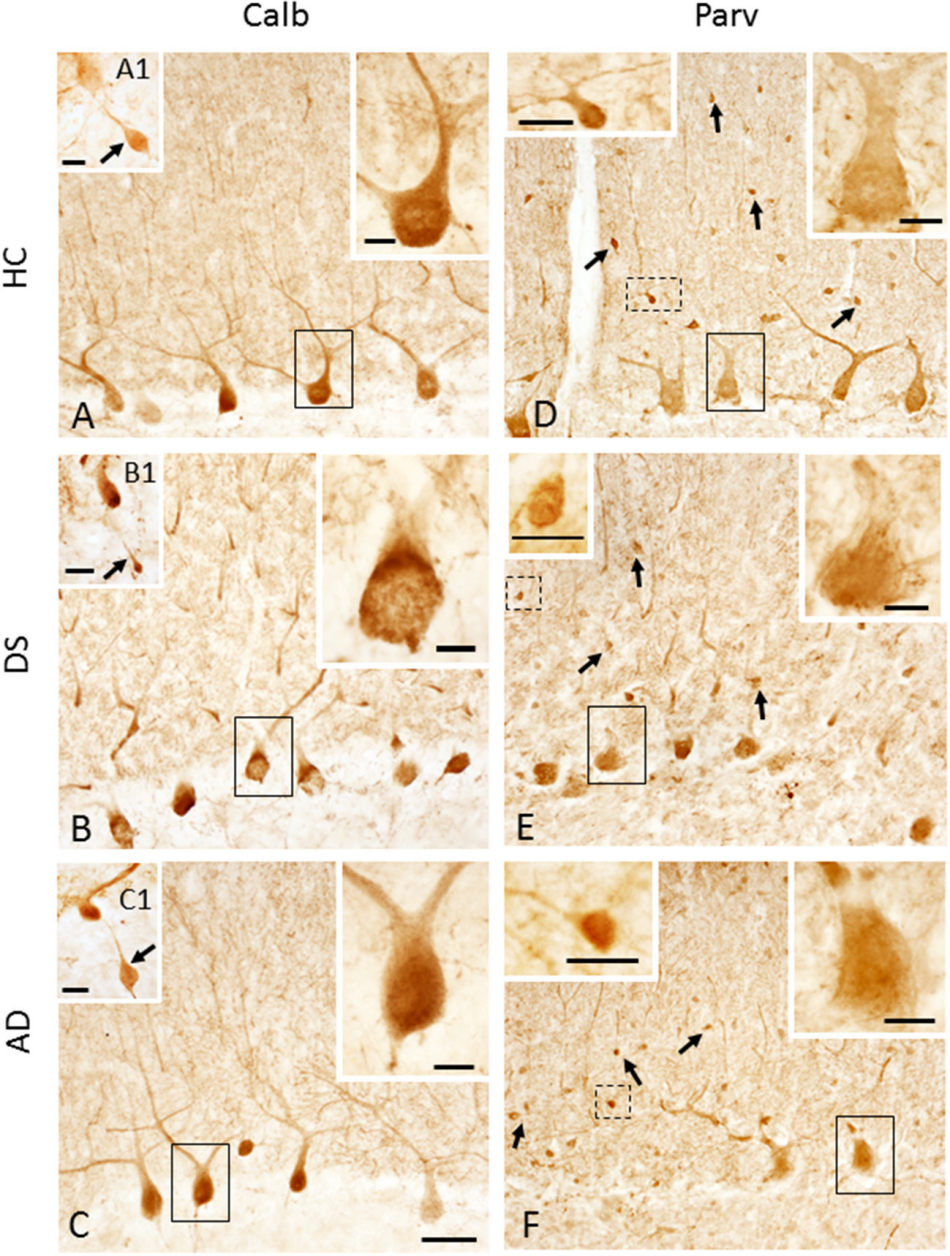
Photomicrographs showing Calb-ir PCs in a 66-year-old female HC **(A)**, 47-year-old female with DSD– **(B)** and a 72-year-old female with AD **(C)** case. Upper right insets show high-power image of the black outlined Calb-ir PCs shown in **(A–C)**. Insets **(A1–C1)** show GL Calb-ir axonal torpedoes (arrows) in a 51-year-old male HC **(A1)**, 60-year-old female with DSD– **(B1)** and a 79-year-old female with AD **(C1)** case. **(D–F)** Parv-ir PCs and Parv-ir interneurons (black arrows) within the ML in a 69-year-old female HC **(D)**, 44-year-old female with DSD– **(E)**, and a 72-year-old female with AD **(F)**. Upper right insets **(D–F)** are higher magnification images of the Parv-ir PCs outlined in solid black boxes, while upper left insets are higher magnification photos of the Parv-ir interneurons (outlined by dashed lines) within the ML, most likely stellate and basket interneurons in **(D–F)**, respectively. Note the presence of many more Parv-ir interneurons in HC **(D)** compared to DS **(E)** and AD **(F)**. Scale bars: **(C)** = 50 μm and applies to **(A,B,D–F)**; **(A1–C1)** insets = 30 μm; larger insets in **(A–F)** = 10 μm; smaller insets in **(D–F)** = 20 μm.

**Figure 8:**
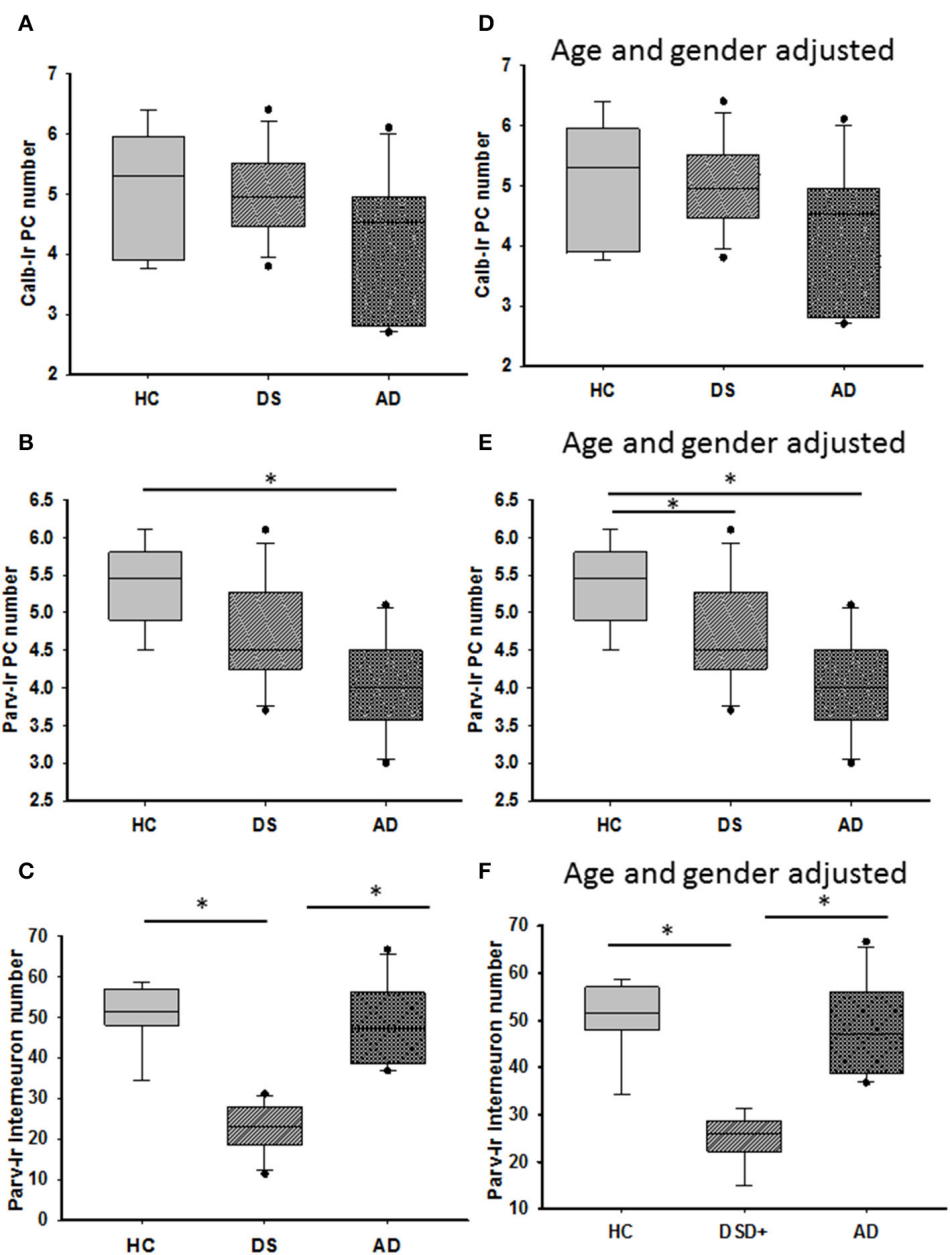
Box plots showing no differences in the number of Calb-ir PCs **(A)** even when adjusted for age and gender **(D)** between the groups. By contrast, HCs had a significantly greater number of Parv-ir PCs compared to subjects with AD (**B**; Kruskal–Wallis, *p* = 0.001). When adjusted for age and gender, HCs had significantly more Parv positive PCs than both subjects with DS and AD (**E**; ANCOVA, *p* < 0.001), while Parv-ir interneurons showed significantly lower numbers in DS compared to HC and AD cases (**C**; Kruskal–Wallis, *p* < 0.001) even after adjusting for age and gender (**F**; ANCOVA, *p* < 0.001). * denotes significance between groups.

Calbindin immunostaining was only seen in PCs (Figures 7A–C, Supplementary Figures 3A,D,G), whereas Parv immunoreactivity was also observed in cells within the ML, most likely stellate and basket interneurons, in each group (Figures 7D–F, Supplementary Figures 3B,E). Small Parv-ir interneurons were less evident in DS (Figure 7E) compared to HC (Figure 7D) and AD (Figure 7F) cases (see also Supplementary Figures 3A–F). Both CBPs were detected in PC soma, proximal and distal dendrites, and axons in HC, DS, and AD cases. However, PC proximal dendrites and axons were less immunoreactive for these CBPs in DS (Figures 7B,E) compared to HC (Figures 7A,D) and AD (Figures 7C,F). Calb-ir axonal torpedoes were observed in HC, DS, and AD subjects (Figures 7A1–C1), while positive dendrites were rare (Supplementary Figures 3G,I). Quantitation revealed no differences in Calb-ir PC number, PC soma and dendritic arborization OD values between groups, with or without DSD– cases (Kruskal–Wallis, *p* > 0.05) (Figure 8A). In contrast, Parv-ir PC numbers were significantly reduced in AD compared to HC (*p* = 0.001) (Figure 8B), while OD values for Parv-ir PC soma were significantly greater in DS than AD (*p* < 0.001). No differences in Parv-ir PC counts or ODs were detected in DS compared to HC cases. However, the number of ML Parv-ir interneurons was significantly reduced in DS compared to HC (Kruskal–Wallis, *p* < 0.001) and AD (Kruskal–Wallis, *p* < 0.001) (Figure 8C). Removal of DSD– cases did not alter these findings. Adjusting for age and gender revealed no statistical differences in number of Calb-ir PC and OD dendritic arborization values among groups, with or without inclusion of DSD– cases (Figure 8D). Counts of Parv-ir PCs revealed a significant reduction in DS and AD compared to the HC group (ANCOVA, *p* < 0.001) independent of DS clinical status (Figure 8E). There were no statistical differences in Parv-ir PC OD values among groups with and without DSD– cases. Parv-ir interneurons were reduced in DS with or without dementia compared to HC and AD cases (Figure 8F) (ANCOVA, *p* < 0.001).

**Figure 9:**
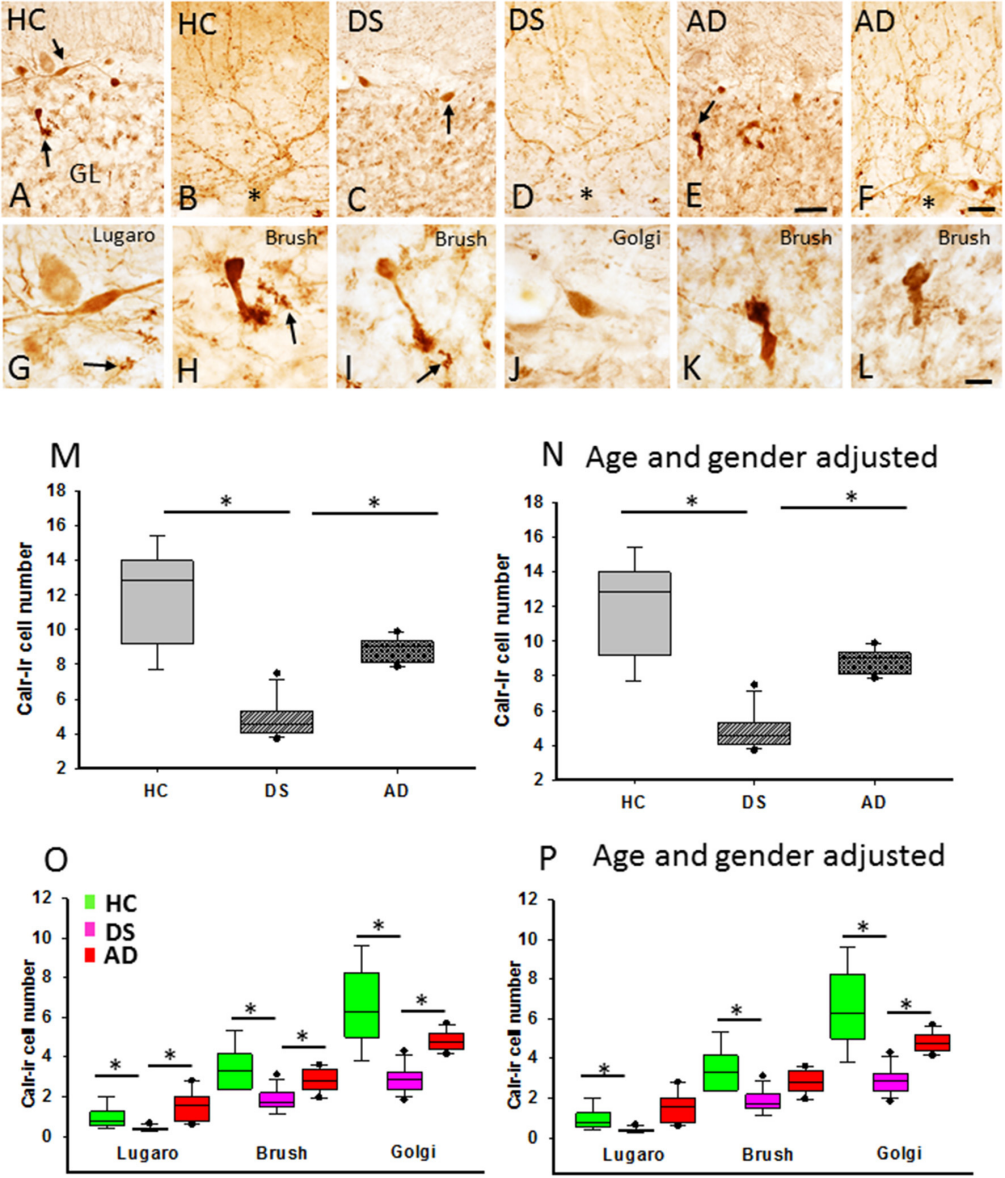
Photomicrographs showing Calr-ir cells **(A,C,E)** and beaded climbing fibers **(B,D,F)** in the GL and ML, respectively, in a 66-year-old female HC **(A,B)**, 46-year-old male with DSD+ **(C,D)**, and a 72-year-old female with AD **(E,F)** case. Note the reduction of Calr-ir cells in DS **(C)** and AD **(E)** compared to HC **(A)**. High-power images of an elongated Lugaro **(G)** shown in **(A)** (upper arrow) and a unipolar brush cell **(H)** (lower arrow in **A**) from a HC, Golgi cell **(J)** shown in **(C)** (arrow) and a unipolar brush interneuron in DS **(I)**, as well unipolar brush cells (**K**: indicated by an arrow in **E**; and **L**) in an AD case. Note the presence of GL Calr-ir MF rosettes in **(G–I)** (arrows). Boxplots showing a significant reduction in Calr-ir cell numbers in DS compared to HC (**M**; Kruskal–Wallis, *p* < 0.001) and AD (**M**; Kruskal–Wallis, *p* < 0.002), even after adjusting for age and gender (**N**; ANCOVA, *p* > 0.005). The number of Calr-ir Golgi, unipolar brush, and Lugaro cells was significantly reduced in DS compared to HC (**O**; Golgi and brush: Kruskal–Wallis, *p* < 0.01; Lugaro: Krukal–Wallis, *p* < 0.001) and AD (**O**; Golgi and brush: Kruskal–Wallis, *p* < 0.01; Lugaro: Kruskal–Wallis, *p* < 0.001). Age and gender adjustment revealed a significant reduction in the Golgi, brush, and Lugaro Calr-ir cell number in DS compared to HC, and only Golgi Calr-ir cell numbers were also reduced compared to AD **(P)**. CF, climbing fibers; MF, mossy fibers. Asterisks in (**B,D,F**) indicate PCs. Scale bars: **(E)** = 50 μm applies to **(A,C)**, **(F)** = 25 μm applies to **(B,D)**, and **(L)** = 10 μm applies to **(G–K)**. * denotes significance between groups.

Calretinin profiles were found in the GL and ML in all cases examined (Figures 9A–L). Calr-ir neurons in the GL displayed features indicative of Lugaro (Figure 9G), unipolar brush (Figures 9H,I,K,L), and Golgi (Figure 9J) interneurons (Diño et al., 1999; Stepień et al., 2012). Calr-ir beaded fibers were seen in close proximity to PC dendrites (Figures 9B,D,F) and forming rosettes in the GL (Figures 9G–I), likely corresponding to cerebellar climbing and mossy fibers (Rogers, 1989; Álvarez et al., 2008), respectively, in all three groups (Figures 9B,D,F,G–I). Brush cells showed stronger Calr immunoreactivity compared to Lugaro and Golgi interneurons (Figures 9A,C,E,G–L). In contrast to HC and AD subjects, Calr-ir cell types were less numerous in DS (Figures 9A,C,E). Counts revealed a significant reduction in the number of total GL Calr-ir cells in DS compared to HC (Kruskal–Wallis, *p* < 0.001) and AD (Kruskal–Wallis, *p* < 0.002) (Figure 9M). Furthermore, the numbers of Calr-ir Golgi, Lugaro, and brush cells were also significantly decreased in DS compared to HC (Golgi and brush: Kruskal–Wallis, *p* < 0.001; and Lugaro: Kruskal–Wallis, *p* = 0.01) and AD (Golgi and brush: Kruskal–Wallis, *p* < 0.01; and Lugaro: Kruskal–Wallis, *p* < 0.001) (Figure 9O). Similar results were obtained when DSD– cases were eliminated from the analysis. Adjusting for age and gender showed a significant decrease in both the total number of Calr-ir interneurons and Calr-ir Golgi cells in DS compared to AD and HC (ANCOVA, *p* < 0.001), while the number of Lugaro (ANCOVA, *p* = 0.04) and brush (ANCOVA, *p* = 0.005) Calr containing cells were greater in HC compared to DS with or without dementia (Figures 9N,P) but not AD. Calr-positive Lugaro cell numbers were not significantly different between HC and DS even when DSD– cases were removed from the analysis.

### Quantitation of p75^NTR^ and TrkA-Positive PCs

**Figure 10:**
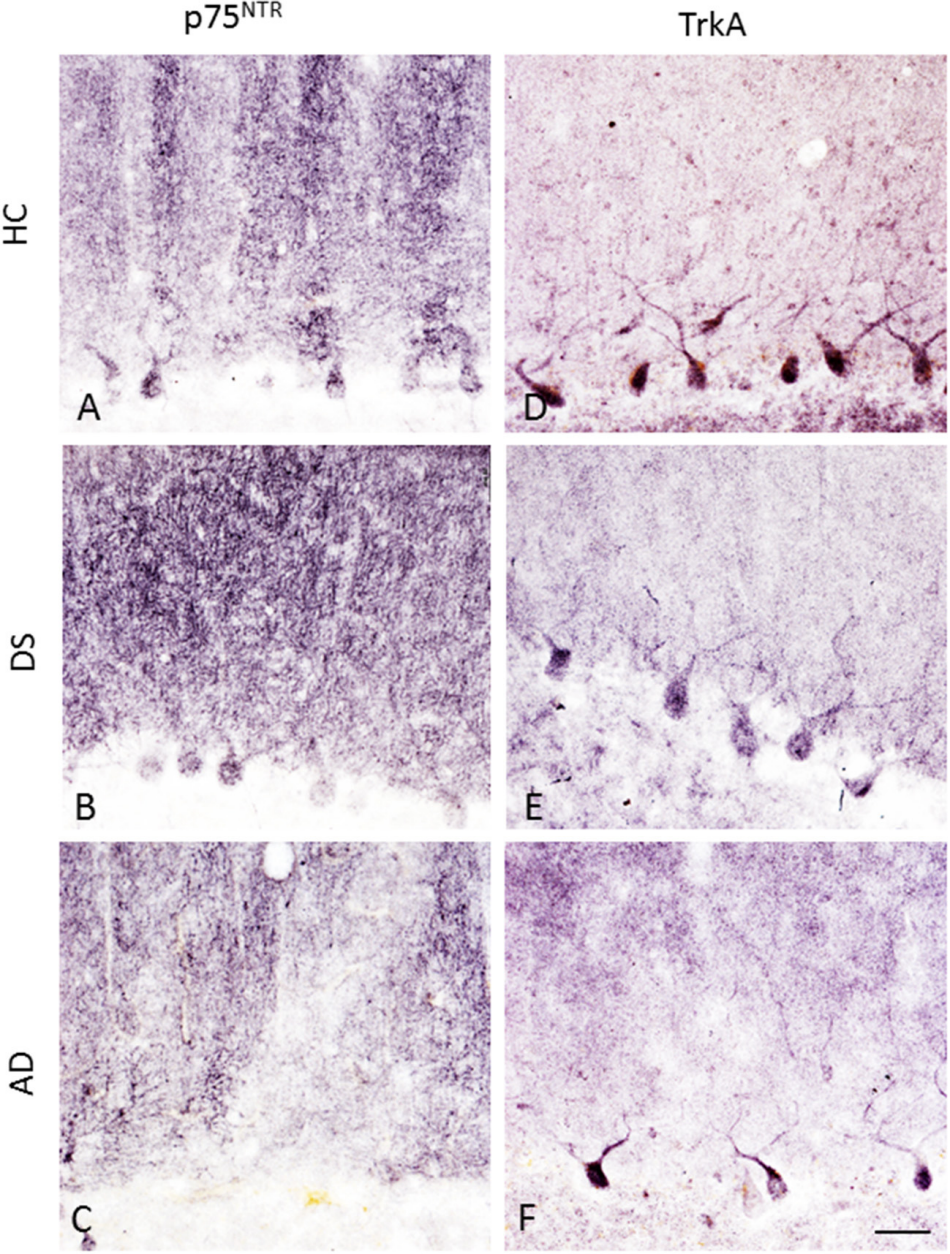
Photomicrographs showing p75^NTR^-ir PC soma, dendritic arbors and axons in a 66-year-old female HC **(A)**, 47-year-old female with DSD– **(B)**, and a 79-year-old female with AD **(C)** case. In contrast, TrkA immunostaining was mainly seen in PC soma with a few positive dendritic branches in a 84-year-old female HC **(D)**, 47-year-old female with DSD– **(E)**, and a 83-year-old female with AD **(F)** case. Scale bar = 50 μm in **(A–F)**.

Purkinje cells soma displayed p75^NTR^ and TrkA immunoreactivity across all the groups (Figure 10). Although PC p75^NTR^ dendritic trees extended into ML, similar TrkA-positive profiles were not observed in HC (Figure 10D), DS (Figure 10E) or AD (Figure 10F). P75^NTR^ but not TrkA-ir torpedoes were seen in the GL in DS, AD, and HC subjects (data not shown).

**Figure 11:**
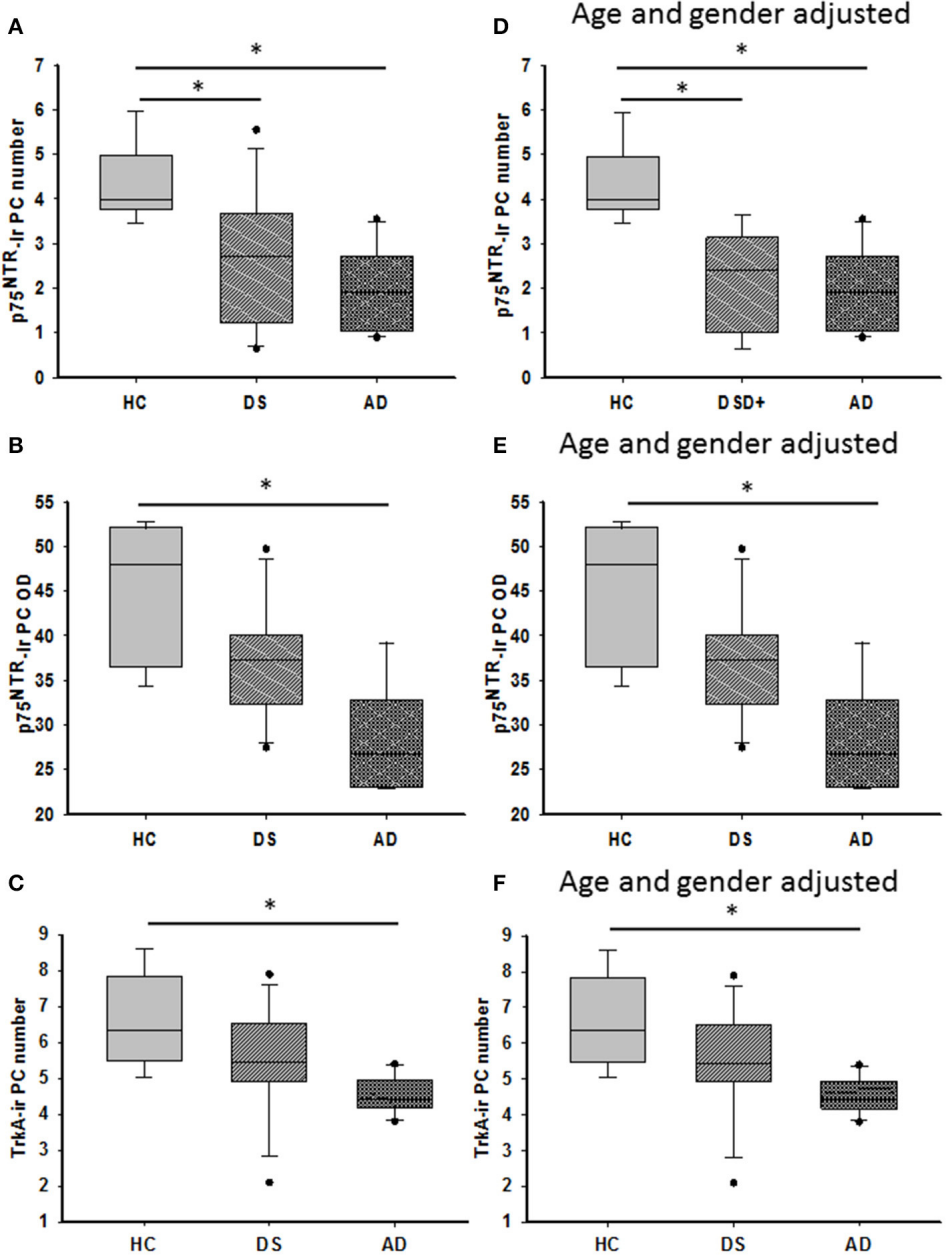
Boxplots showing a significant reduction in the number of p75^NTR^ positive PCs in both DS (**A**; Kruskal–Wallis, *p* = 0.03) and AD (**A**; Kruskal–Wallis, *p* = 0.001) compared to HC. Adjusting for age and gender revealed statistically similar results when DSD– cases were removed from the analysis (**D**; ANCOVA, *p* < 0.011). Optical density (OD) measurements of p75^NTR^-ir PC soma revealed significantly higher values in HC compared to AD (**B**; Kruskal–Wallis, *p* = 0.009) but not different from subjects with DS. Similar statistical findings were found when adjusted for age and gender (**E**; ANCOVA, *p* < 0.011). Boxplots revealed a significant reduction in the number of TrkA-ir PCs in AD compared to HC but not in DS (**C**; Kruskal–Wallis, *p* < 0.05) even after adjusting for age and gender (**F**; ANCOVA, *p* < 0.001). * denotes significance between groups.

Quantitation showed higher numbers of TrkA- compared to p75^NTR^-ir PCs in all the groups (Mann–Whitney, *p* ≤ 0.001). Counts between groups revealed a significant reduction in the number of p75^NTR^-ir PCs in both AD (Kruskal–Wallis, *p* = 0.001) and DS (Kruskal–Wallis, *p* = 0.03) compared to HC (Figure 11A). When DSD– cases were removed from the analysis, the significance of this reduction was increased in DS compared to HC (Kruskal–Wallis, *p* = 0.004). The OD measurement of PC soma immunoreactive for p75^NTR^ was significantly higher in HC compared to subjects with AD (Kruskal–Wallis, *p* = 0.009) (Figure 11B) but not different from DS independent of phenotype. By contrast, OD measurement of the PC dendritic tree displaying p75^NTR^ immunoreactivity revealed no significant difference between DS groups with or without dementia (Kruskal–Wallis, *p* > 0.05). The number of TrkA-positive PCs was significantly lower in AD compared to HC but not DS (Kruskal–Wallis, *p* < 0.05) (Figure 11C) with or without DSD– cases. TrkA-positive PC OD measurements showed a trend toward a decrease in AD and DS compared to HC subjects but did not reach significance when analyzed with (Kruskall–Wallis, *p* = 0.065) or without DSD– (Kruskall–Wallis, *p* = 0.059) cases.

Adjusting for age and gender revealed that the number of p75^NTR^ and TrkA-positive PCs was significantly reduced in AD compared to HC (ANCOVA, *p* < 0.011) (Figures 11D,F), but no difference was found in the DS cases. In addition, p75^NTR^, but not TrkA-positive PC number was significantly decreased in DS compared to HC when DSD– cases were eliminated from the analysis (ANCOVA, *p* < 0.001) (Figure 11D). P75^NTR^-ir PC perikaryon, but not dendritic arborization OD values were significantly greater in HC compared to AD (ANCOVA, *p* = 0.009) (Figure 11E) but not DS. A sub-analysis removing the DSD– cases revealed similar results (ANCOVA, *p* = 0.004).

### Correlations Between Cerebellar Neuron Counts and Case Demographics

**Figure 12:**
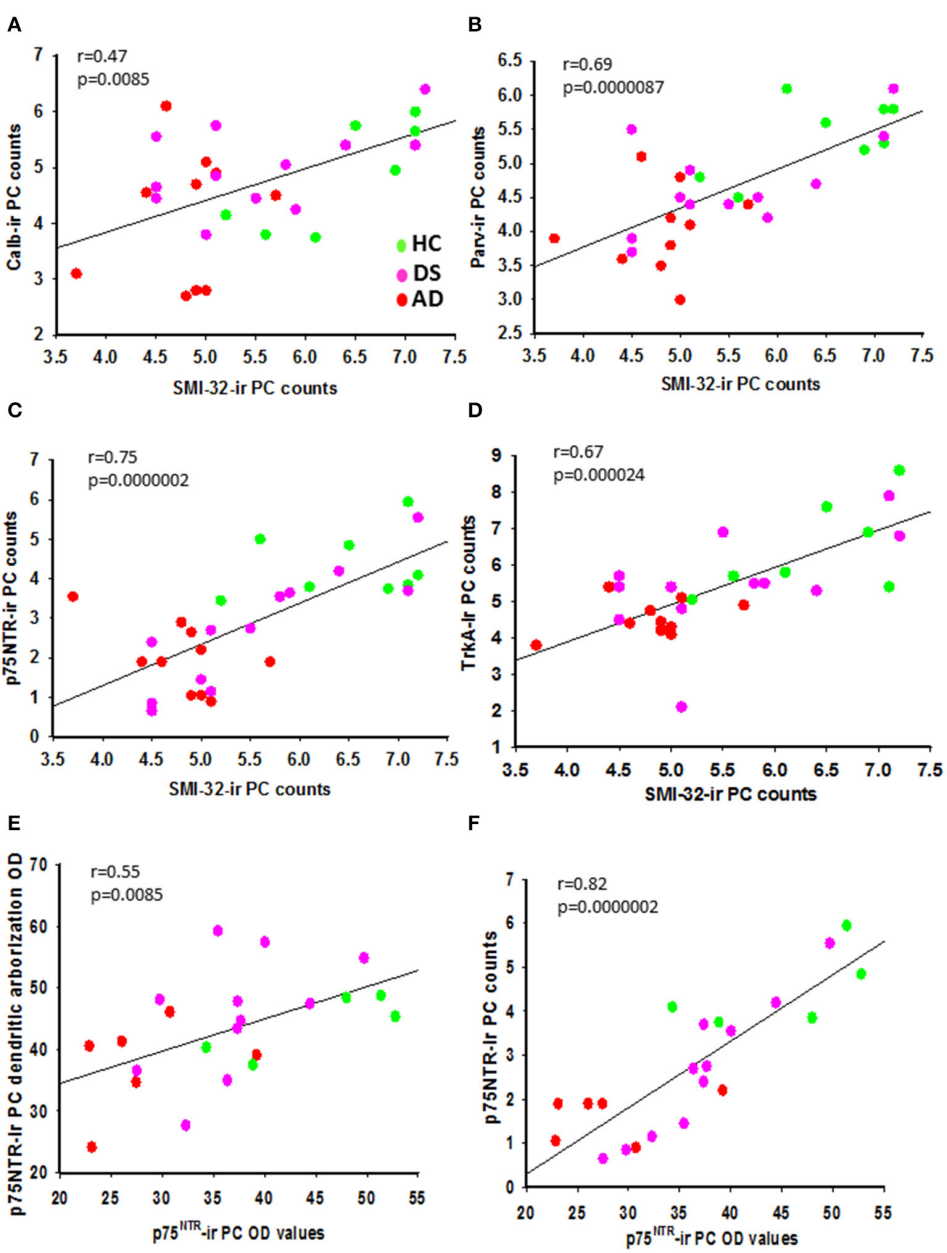
Linear regression analysis revealed significant positive correlations between SMI-32 and Calb- (**A**; *r* = 0.47, *p* = 0.085), Parv- (**B**; *r* = 0.69, *p* 0.0000087), p75^NTR^- (**C**; *r* = 0.75, *p* = 0.0000002), and TrkA-ir PC counts (**D**; *r* = 0.67; *p* = 0.000024) across the three groups. Significant positive correlations were seen between p75^NTR^-ir PC soma and p75^NTR^-ir PC dendritic arborization OD values (**E**; *r* = 0.55; *p* = 0.0085) and p75^NTR^-ir PC counts (**F**; *r* = 0.82; *p* = 0.0000002) across groups.

We found positive correlations between H&E and cresyl violet stained PC number (*r* = 0.482, *p* = 0.007) and GL and ML thickness (*r* = 0.752, *p* = 0.0000002) across the groups. A significant positive correlation was found between APP/Aβ and Aβ_42_ plaque load (*r* = 0.838, *p* = 0.0000002) within the ML. Number of PC SMI-32-positive neurons correlated positively with Parv-ir (Figure 12B; *r* = 0.69, *p* = 0.0000087), p75^NTR^-ir (Figure 12C; *r* = 0.075, *p* = 0.0000002), TrkA-ir (Figure 12D; *r* = 0.67, *p*= 0.000024), and cresyl violet stained neurons in this region, but to a lesser degree with Calb-ir PC counts (Figure 12A; *r* =0.47, *p* = 0.0085) across the groups. In addition, we found a positive correlation between PC TrkA-ir number and TrkA-ir PC soma OD measurements (Supplementary Figure 4B; *r* = 0.57, *p* = 0.0053). However, no significant correlations were found between PC TrkA and p75^NTR^ soma OD values. There was a correlation between the number of Parv and Calb PCs and TrkA and p75^NTR^ containing PCs. Parv-ir PC numbers were not correlated with Parv PC soma OD values. SMI-32 and Parv PC soma OD values were positively correlated (*r* = 0.424, *p* = 0.02), but there was no correlation with the number of SMI-32 positive PCs. There was a positive correlation between OD values for p75^NTR^ PC soma (Figure 12F; *r* = 0.82, *p* = 0.0000002), p75^NTR^ dendritic arborization OD values (Figure 12E; *r* = 0.55, *p* = 0.0085), and SMI-32-ir PC counts (Supplementary Figure 4A; *r* = 0.68, *p* = 0.00038) across the groups. There was a strong negative correlation between the number of p75^NTR^, but not TrkA-positive PCs and APP/Aβ plaque load in AD (*r* = −0.923, *p* = 0.0000002), but not in DS (*r* = 0.35, *p* = 0.2) or across groups (*r* = −0.37, *p* = 0.06). Interestingly, the numbers of GL SMI-32- and SMI-34-ir torpedoes were positively correlated between AD and HC (*r* = 0.60, *p* = 0.007) but not across all cohorts. SMI-34-ir torpedoes correlated negatively with Aβ_42_ plaque load (*r* = −0.60, *p* = 0.0007) but not with APP/Aβ plaque load across the groups. Furthermore, Parv-ir stellate/basket cell counts correlated strongly with total number of Calr-ir brush, Lugaro, and Golgi interneurons (Supplementary Figure 4C; *r* = 0.76, *p* = 0.0000002). Golgi cell numbers correlated with Calr positive brush (Supplementary Figure 4E; *r* = 0.73, *p* = 0.0000002), Lugaro (Supplementary Figure 4F; *r* = 0.68, *p* = 0.00003), and Parv-positive interneuron counts (Supplementary Figure 4D; *r* = 0.74, *p* = 0.0000002) across the groups. Parv-ir interneuron counts showed a negative association with OD values for SMI-32 PC soma (*r* = −0.57, *p* < 0.001), Parv-ir neurons (*r* = −0.48, *p* = 0.008), Aβ_42_ plaque load (Supplementary Figure 5A; *r* = −0.74, *p* = 0.0000002), and APP/Aβ (Supplementary Figure 5B; *r* = −0.61, *p* = 0.00033) plaque load. We also found negative correlations between the number of GL Calr positive cells, Aβ_42_ plaque load (Supplementary Figure 5C; *r* = −0.70, *p* = 0.0000002), and APP/Aβ plaque load (Supplementary Figure 5D; *r* = −0.47, *p* = 0.0082) across the groups. Calr-ir Lugaro cell numbers correlated negatively with Parv-ir PC OD values (*r* = −0.60, *p* < 0.001). Number of Calr-ir brush cells correlated positively with the number of p75^NTR^ PCs (*r* = 0.50, *p* < 0.008) across groups. In addition, we found strong negative correlations between Calr-ir Golgi and brush cell counts (*r* = −0.65, *p* = 0.00013) with Aβ_42_ plaque load (Supplementary Figure 5E; *r* = −0.71, *p* = 0.0000002) but a weaker association with APP/Aβ plaque load (Supplementary Figure 5F; *r* < −0.5 and *p* = 0.014), while Calr-ir Lugaro counts correlated only with Aβ_42_ plaque load (*r* = −0.52, *p* = 0.003).

Aβ_42_ plaque load correlated negatively with age (*r* = −0.630, *p* = 0.0002). Calb (*r* = −0.429, *p* = 0.018) and Parv (*r* = −0.397, *p* = 0.03) positive PC counts displayed a weak negative association with age across groups. OD values for Parv positive PCs showed a strong negative association with increased age (*r* = −0.705, *p* = 0.000002). These age-related negative correlations are most likely due to the significantly younger age of the DS compared to the AD and HC subjects. GL SMI 34-ir, but not SMI-32 torpedo counts positively correlated with age (Supplementary Figure 6A; *r* = 0.62, *p* = 0.0003). Number of SMI-32 (Supplementary Figure 6B; *r* = −0.66 *p* = 0.00007), p75^NTR^ (Supplementary Figure 6C; *r* = −0.73, *p* = 0.0000002), Parv (Supplementary Figure 6D; *r* = −0.59, *p* = 0.00064), and TrkA (Supplementary Figure 6E; *r* = −0.57, *p* = 0.001)-positive PC counts, but not Calb cells, showed a significant negative association with Braak NFT scores across groups. There was a negative correlation between Braak scores and both p75^NTR^ (Supplementary Figure 6F; *r* = −0.57, *p* = 0.006) and TrkA (Supplementary Figure 7A; *r* = −0.56, *p* = 0.008) PC soma OD values as well as Calr positive cell counts (Supplementary Figure 7B; *r* = −0.58, *p* = 0.00094).

## Discussion

Down syndrome is characterized, in part, by cognitive impairment, which is present to some degree in all people with this disorder (Pennington et al., 2003). By age 40, all individuals with DS exhibit NFT and amyloid lesions similar to those observed in the AD brain with a concentration in neo and limbic cortical regions (Leverenz and Raskind, 1998; Head et al., 2012; Davidson et al., 2018; Perez et al., 2019), and display impairments in learning, memory, language, and motor behaviors (Rajmohan and Mohandas, 2007; Rolls, 2015). Neuropathological examination has shown a significant reduction in cerebellar volume/size in infants, children, and adults with DS (Guidi et al., 2011; Mufson et al., 2020) that is recapitulated in genetic mouse models of this disorder (Necchi et al., 2008; Lomoio et al., 2009). Although the cerebellum plays a key role in the regulation of proprioceptive-motor control and motor learning (Spanò et al., 1999; Malak et al., 2015), evidence suggests that it is also involved in higher order functions including cognition (Schmahmann, 2004), dysmetria of thought (Schmahmann, 1991), and the cerebellar cognitive affective syndrome (Tavano et al., 2007). Cerebellar lesions can lead to the development of a behavioral pattern characterized by reduced cognitive efficiency associated with executive and visuospatial, expressive language, and affective disorders (Tavano et al., 2007; Yildiz et al., 2010). Despite data linking cerebellar dysfunction to defects in cognition, there are virtually no detailed investigations of the cellular pathobiology of the adult cerebellum in individuals with DS. Here we present, a first of its kind report describing alterations in PCs and interneurons that contain either CBPs or the cognate NGF receptors, TrkA, and p75^NTR^ and their relation to AD-like pathology in the cerebellum of older people with DS compared to AD and HC.

Amyloid plaques and NFTs are common pathological manifestations that appear in most adults with DS before the age of 50 (Mann and Esiri, 1989). In the present study, we observed APP/Aβ and Aβ_42_ positive cerebellar plaques and blood vessels as well as Aβ_40_ leptomeningeal vessels, but not Aβ_40_ plaques, or NFTs in dementia and non-dementia DS cases (Tamaoka et al., 1995; Mann et al., 1996; Wang et al., 2002). Cerebral angiopathy was more frequently seen in the cerebellar cortex in DS than AD suggesting a more compromised vasculature in DS. Within cerebellar cortex, diffuse APP/Aβ and Aβ_42_ deposits were mainly found in the ML, with few scattered APP/Aβ deposits in the GL, PC layer, and white matter in both DS (Cole et al., 1993; Li et al., 1994; Cataldo et al., 1996; Mann et al., 1996) and AD (Pro et al., 1980; Joachim et al., 1989; Ogomori et al., 1989; Lemere et al., 1996; Wang et al., 2002; Mavroudis et al., 2010; Sepulveda-Falla et al., 2014; Catafu et al., 2016; Jacobs et al., 2018). Aβ_42_ and APP/Aβ plaque loads were significantly greater in DS compared to HC, while only Aβ_42_ plaque load was increased in DS compared to AD. When adjusted for age and gender DS still displayed a greater Aβ_42_ plaque load than HC and AD with no difference in APP/Aβ plaque load among groups, suggesting that Aβ_42_ plaques in the cerebellum develop at different rates in DS than in AD. Likewise, the ratio of Aβ_42_:APP/Aβ plaque load was greater in DS compared to AD suggesting an accelerated production of Aβ_42_ or higher levels of Aβ N-terminal truncation that precludes recognition by the APP/Aβ 6E10 antibody (Kummer and Heneka, 2014; Thal et al., 2015) a major Aβ component in the cerebellum in DS (Lalowski et al., 1996). However, adjusting for age and gender revealed no significant difference in Aβ_42_:APP/Aβ plaque ratio between groups. The observation that the DS cerebellum contains early stage diffuse non-neuritic plaques (Thal et al., 2006; Catafu et al., 2016) with a filamentous appearance compared to more advanced neuritic plaques reported in the neocortex of both DS and AD (Hof et al., 1995; Nelson et al., 2012; Perez et al., 2019), suggests regional differences in plaque development between these disorders. Unlike sporadic AD (Braak and Braak, 1997; Thal et al., 2002; Verdile et al., 2004), DS amyloid cerebellar plaques remain diffuse even at older ages, suggesting that trisomy APP overexpression differentially affects plaque formation and maturation within different brain regions in DS. Perhaps the lack of neuritic core plaques plays a role in the absence of tau pathology in the cerebellum in DS (present findings; Mann and Jones, 1990; Cole et al., 1993; Li et al., 1994; Davidson et al., 2018) and AD (Aikawa et al., 1985; Azzarelli et al., 1985; Joachim et al., 1989; Mann and Jones, 1990; Li et al., 1994; Mann et al., 1996; Zhu et al., 2019). Interestingly, the morphology and location of amyloid plaques seen in the DS cerebellum is similar to that described in early-onset familial AD caused by a genetic mutation of the Presenilin-1E280A gene (Sepulveda-Falla et al., 2012).

Quantitation of cerebellar PCs stained for H&E or cresyl violet was similar across groups even when adjusted for age and gender supporting prior findings showing no difference in PC soma numbers between AD and HC cases (Andersen et al., 2012; Stepień et al., 2012; Mavroudis et al., 2013, 2019; Tabatabaei-Jafari et al., 2017). Surprisingly, we found a significantly higher number of H&E compared to cresyl violet stained PCs in DS and AD but not in HC cases. Perhaps, this disparity is related to alterations in the cellular milieu underlying the affinity of each histochemical stain. In this regard, cresyl violet labels mRNAs located in Nissl bodies, whereas H&E stains cytoplasm, nuclei, and organelles. The present results derived from the cresyl violet staining suggest mRNA defects in PCs in DS and AD that are not found in HCs. These findings also suggest that there is not a frank loss of PCs in the cerebellum of adults with DS. By contrast, others using cresyl violet and H&E stains report a significant decrease in PC density in AD compared to controls (Fukutani et al., 1996; Wegiel et al., 1999; Sjöbeck and Englund, 2001; Mavroudis et al., 2010), and that PC loss in familial AD (FAD) was greater than in sporadic AD (Fukutani et al., 1997). The discrepancy between the present and earlier findings may be due to a variation in counting procedures and/or cohort. By contrast, we found a highly significant decrease in mean number of SMI-32-ir PCs in DS and AD compared to HC, when adjusted for age and gender. SMI-32 is a well-characterized antibody raised against non-phosphorylated-high-molecular weight neurofilament proteins (NFH). Neurofilament proteins are cytoskeletal polymers found predominantly in axons that are essential for axonal maintenance and rate of action potential propagation (Sternberger and Sternberger, 1983; Burianová et al., 2015). While our study appears to be the first to describe a decrease in SMI-32 in the cerebellum of an adult with DS, similar decreases are reported in the cortex (Morrison et al., 1987; Hof and Morrison, 1990; Hof et al., 1990; Bussière et al., 2003; Ayala-Grosso et al., 2006; Thangavel et al., 2009) and the hippocampus in AD (Cork et al., 1986; Vickers et al., 1992, 1994; Thangavel et al., 2009) as well as in the brains of normal aged humans (Vickers et al., 1994). Reductions in SMI-32 staining in cortical neurons are associated with an increase in phosphorylation of the neurofilaments that contribute to NFT formation in AD (Cork et al., 1986; Hof et al., 1990; Morrison and Hof, 2002; Veeranna et al., 2011; Vickers et al., 2016). Unlike SMI-32, which reveals non-phosphorylated NFH in PCs, none were SMI-34 positive for phosphorylated high molecular weight neurofilaments in DS, AD, or HC. However, it is known that non-phosphorylated NFH epitopes become increasingly phosphorylated during the aging process (Burianová et al., 2015) and abnormal hyperphosphorylation is considered a trigger for neurofilament accumulation associated with neurodegeneration (Petzold, 2005). Here, we found a greater number of phosphorylated compared to non-phosphorylated NFH torpedoes in DS, AD, and HC subjects. Moreover, the number of GL phosphorylated NFH torpedoes was significantly increased in AD compared to DS, which correlated positively with age and negatively with Aβ plaque load across groups, suggesting aging, but not Aβ pathology, as a factor in the formation of phosphorylated NFH torpedoes. PC axonal torpedoes, which consist of disordered phosphorylated and non-phosphorylated neurofilaments, are thought to underlie defects in axonal transport (Jung et al., 2000; Cleveland and Rothstein, 2001; Robertson et al., 2002; Liem and Leung, 2003; Louis et al., 2012; Didonna and Opal, 2019). The mechanism(s) that trigger PC axonal hyperphosphorylation remain unclear. It is possible that PC torpedoes and hypertrophic axons represent compensatory responses (Kemp et al., 2016) due to neuronal/axonal injury (Petzold, 2005). Together these findings suggest that the reduction of SMI-32 positive PCs and the presence of phosphorylated and non-phosphorylated NFH axonal torpedoes are indicative of alterations in the PC cytoskeleton in DS and AD. Whether PC torpedoes are an age-related phenomenon, a response to injury or driven by genetic or epigenetic factors in DS and AD requires further investigation. Interestingly, alterations in cerebrospinal fluid and blood neurofilament levels are potential diagnostic/prognostic biomarkers for neurodegenerative diseases, including DS (Rafii et al., 2015; Strydom et al., 2018; Fortea et al., 2020) and AD (Jin et al., 2019; Raket et al., 2020).

Purkinje cells are phenotypically characterized by Calb and Parv. These CBPs regulate calcium levels either directly or indirectly enabling (de)sensitization of calcium channels controlling calcium entry into cells to maintain cerebellar function (Bastianelli, 2003). The present study found no difference in the number of Calb immunolabeled PCs and OD values among groups supporting previous report (Stepień et al., 2012). By contrast, a loss of Calb-ir neurons has been reported in the hippocampus (McLachlan et al., 1987; Stefanits et al., 2014), cerebral cortex (Ichimiya et al., 1988) and nucleus basalis of Meynert (Ichimiya et al., 1989; Riascos et al., 2011) in AD. In addition, there is an age-related decrease in PC Calb protein and mRNA levels in humans (Iacopino and Christakos, 1990; Gattoni and Bernocchi, 2019) and rodents (Iacopino and Christakos, 1990; Amenta et al., 1994; Kishimoto et al., 1998). The present findings revealed age as a cofactor underlying changes in Calb containing PCs. Functionally, neuronal expression of Calb confers resistance to neurodegenerative processes during normal aging (Geula et al., 2003) and AD (Riascos et al., 2011). For example, Calb-containing cholinergic forebrain neurons are resistant to phosphorylated tau accumulation and tangle formation in AD (Riascos et al., 2011). In contrast, Parv-ir PC counts were significantly reduced in DS (with and without dementia) and in AD compared to HC cases when adjusted for age and gender (present study). Furthermore, we found fewer ML Parv-ir interneurons (stellate and basket cells) in DS than in AD and HC cases. Since Parv-positive PCs, stellate and basket cells also contain the inhibitory neurotransmitter GABA (Schwab et al., 2013), it is possible that these neurons are more vulnerable to pathological insults in DS. Unlike Calb, no differences in cerebellar Parv-ir neuron number and expression were reported between the young and elderly humans (Satoh et al., 1991), suggesting the decrease in Parv-ir PCs is not age-dependent. However, a reduction in PC Parv immunoreactivity (Stepień et al., 2012) and cell size (Satoh et al., 1991) has been described in AD compared to controls. Decreases in PC Parv mRNA levels and Parv-ir neuron numbers occur in people with autism (Soghomonian et al., 2017), while an increase in cerebellar Parv levels were found in the schizophrenic brain (Vidal-Domènech et al., 2020). These observations indicate that Parv cells are vulnerable in various neurologic disorders, whereas Calb is associated with cellular resistance in the face of neuropathologic diseases (Fairless et al., 2019).

While Calr shares extensive homologies with Calb, the former is observed in a separate population of neurons in the cerebellar cortex. Here, we found Calr positive GABAergic inhibitory Golgi and Lugaro and excitatory unipolar brush cells only in the granular cell layer of the cerebellum. Brush cells are classified in two functionally and chemically distinct subclasses: Type I contain Calr, while Type II are characterized by expression of mGluR1α (Martina and Sekerková, 2016). Based upon these phenotypes, most of the brush cells described here are likely Type I. We found a significant reduction in all three Calr containing cell types in DS compared to HC, while no differences were observed between AD and HC cases. There was a strong correlation between these interneuronal subtypes and Parv-ir interneurons within the ML, possibly due to their close synaptic interrelationships. Brush cells establish contacts upon afferent mossy fibers, which are modulated by the Golgi cells, and send apical dendrites to the ML, which receive inputs from the basket and Lugaro cells (Geurts et al., 2003). Furthermore, a strong association was found between Aβ_42_ plaque load in the ML and low numbers of Parv- and Calr-ir Golgi interneurons across groups suggesting a possible neurotoxic amylogenic effect. However, human and animal studies suggest that Calr and Calb provide resistance to tau and beta amyloid pathology (Riascos et al., 2011). Since cerebellar PCs contain both Calb and Parv (Baimbridge et al., 1992), this population may be less susceptible to calcium dehomeostasis and neurodegeneration (Arbel-Ornath et al., 2017). We found a reduction in Parv-positive PCs in DS and AD compared to HC and a loss of Parv- and Calr-ir interneurons in DS compared to AD and HC, suggesting that cerebellar CBP circuits are more compromised in DS than AD. Interestingly, Nanostring genomic data derived from the frontal cortex of adults with DS revealed significant decreases in Parv and Calr transcripts compared to HC and AD (unpublished data). At what stage CBP defects occur during the development of the DS brain requires further investigation. Overall, the present data suggest that neuronal calcium dysregulation plays a role in GABAergic inhibitory neurotransmission in DS.

Cerebellar PCs are also characterized by the expression of the neurotrophin protein NGF (Shelton and Reichardt, 1986) and its cognate low affinity p75^NTR^ and high affinity TrkA receptors (Cohen-Cory et al., 1991; Mufson et al., 1991; Hock et al., 1998; Triaca et al., 2016). NGF binds to its TrkA receptor, activating signal transduction pathways key for neuronal survival (Kaplan and Miller, 2004), while p75^NTR^, a modulator of NGF/TrkA binding (Kaplan and Miller, 2004), is associated with cell death (Mufson et al., 2019). Our quantitative analysis revealed a significant reduction in the number of TrkA-ir PCs in AD compared to HC but not DS. AD cases displayed a 31% and DS a 14% reduction in TrkA-positive PCs compared to HC. By contrast, there were 47% fewer TrkA-ir neurons within nucleus basalis of subjects with DS than aged controls (Sendera et al., 2000). We also found a significant reduction in the number of p75^NTR^-ir PCs in AD and DS compared to HC cases. P75^NTR^ PC soma OD measurements were significantly reduced in AD compared to HC but not in DS. However, when the data were adjusted for age and gender, the number of TrkA and p75^NTR^ containing PC cells decreased in AD compared to HC but only p75^NTR^ PCs in DSD+ compared to HC. By contrast, it has been reported that TrkA and p75^NTR^ mRNA levels are not altered in the AD cerebellum compared to controls (Hock et al., 1998). Discrepancy between protein and mRNA is frequently reported and could explain these contradictory findings (Gygi et al., 1999; Washburn et al., 2003). Although we did not find an association between cerebellar APP/Aβ and Aβ_42_ load with numbers of TrkA or p75^NTR^ labeled PCs in DS, a strong negative relationship was observed between APP/Aβ load and p75^NTR^ but not in the number of TrkA containing PCs in AD. TrkA binds APP preventing its cleavage into Aβ peptides (Costantini et al., 2005; Triaca et al., 2016) and activated Trks suppress apoptotic pathways induced by the binding of Aβ to p75^NTR^ (Matrone et al., 2008, 2009, 2011). Perhaps, the genetic overexpression of APP in DS leads to an increase in APP/TrkA binding (Triaca et al., 2016), protecting PCs from apoptosis activated *via* p75^NTR^. Others have reported a lower expression of p75^NTR^ in PC neurons in healthy adults (Shelton and Reichardt, 1986; Koh and Loy, 1989; Cohen-Cory et al., 1991) with re-expression induced post-injury (Sofroniew et al., 2001). These observations together with the present findings showing similar numbers of PCs positive for TrkA and p75^NTR^ in DS, but decreases in AD compared to HC subjects, indicate that cerebellar NGF metabolism is less affected in DS than in AD. Examination of other components of NGF metabolism, which have been studied in DS (Iulita et al., 2014) and AD (Mufson et al., 2019) are required to decipher the molecular mechanisms underlying the role that NGF and its receptors play in PC dysfunction in DS.

**Figure 13:**
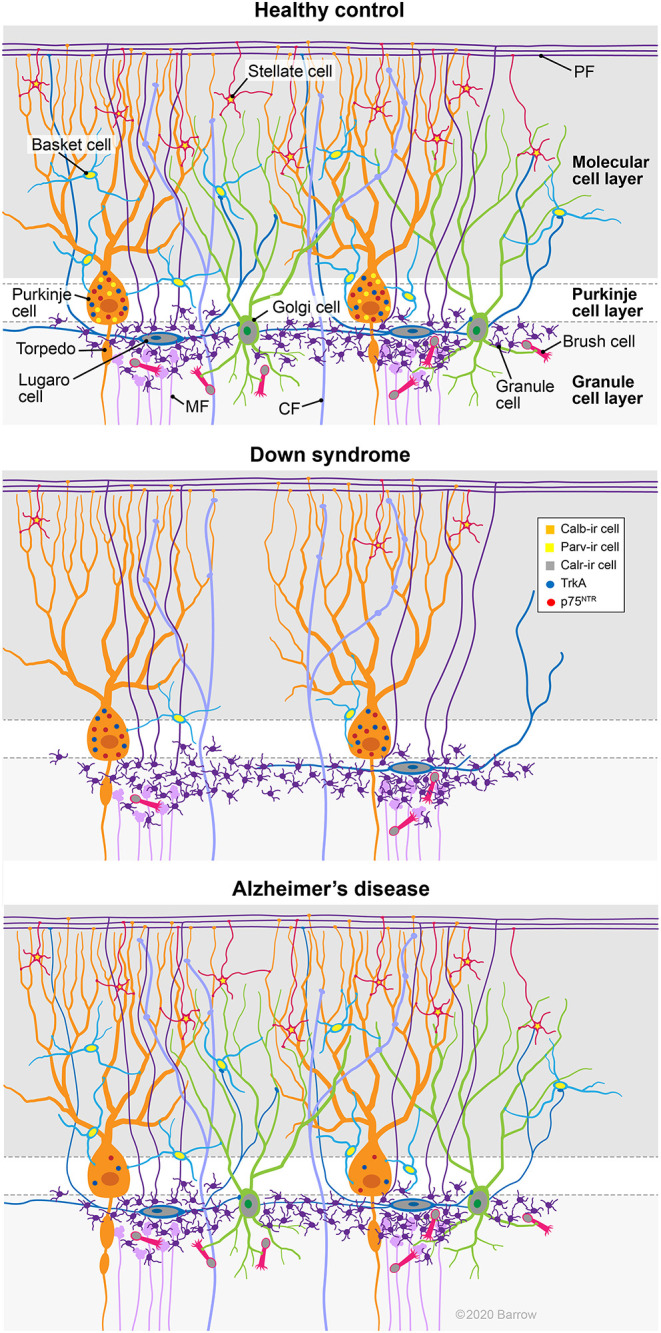
Summary diagram depicts cellular alterations in DS and AD compared to HC cases. Note the reduction of Parv containing PCs as well as Parv (basket/stellate) and Calr (Golgi, Lugaro, and brush) positive interneurons in the cerebellar cortex of an adult with DS. By contrast, TrkA and p75^NTR^-ir PC numbers were lower in AD compared to HC, but not in DS. CF, climbing fibers; MF, mossy fibers; PF, parallel fibers. Used with permission from Barrow Neurological Institute, Phoenix, Arizona.

The findings reported in the present study are summarized in Figure 13. Here, we provide the first evidence of a reduction in Parv containing PCs as well as Parv (basket/stellate) and Calr (Golgi, Lugaro, and brush)-positive interneurons in the cerebellar cortex of adults with DS. In contrast, TrkA and p75^NTR^-ir PC number was lower in AD compared to HC but not in DS. Although, we did not find NFTs and neuritic plaques in the cerebellum of adults with DS, deficits in CBPs and/or NGF metabolism in the cerebellar connectome may play a role in the cognitive and motor deficits reported in DS and AD. Overall, we have shown extensive cellular degenerative events in the cerebellum that should be considered as potential targets for therapeutic intervention in DS and AD.

## Data Availability Statement

The raw data supporting the conclusions of this article will be made available by the authors, without undue reservation.

## Author Contributions

SP and EM: study concept and design, analysis and interpretation of data, and study supervision. JM and MM-A: acquisition of data. JM, SP, and EM: drafting of the article. All authors had full access to all of the data in the study and take responsibility for the integrity of the data and the accuracy of the data analysis.

## Conflict of Interest

The authors declare that the research was conducted in the absence of any commercial or financial relationships that could be construed as a potential conflict of interest.

## Supplementary Material

Supplementary Figure 1 Photomicrographs demonstrating the lack of Purkinje cell (PC) TrkA (**A**, arrows) and p75^NTR^ (**C**, arrows) immunostaining after omission of each antibody compared to antibody reactiviy for TrkA **(B)** and p75^NTR^ **(D)** in a female 47-year-old DSD– case. Lower panels demonstrate that the current TrkA antibody does not immunostain neurons containing TrkB. Similar to previous studies (Mufson and Kordower, 1989; Mufson et al., 1989), cholinergic neurons within the nucleus basalis of Meynert (nbM) are TrkA **(E)** and p75^NTR^ **(F)** immunopositive in tissue from a 93-year-old female HC and a 94-year-old female with AD, respectively. **(G,H)** Images showing TrkA immunopositive (dark blue) neurons in the oculomotor/crainal nerve III (**G**) in contrast to the absence of TrkA containing neurons in substantia nigra (SN) pars compacta (Sobreviela et al., 1994, **H**, arrows), which express TrkB, but not TrkA in tissue obtained from a 51-year-old male HC. Findings support the specificity of the TrkA immunostaining of PCs shown in the present study. Sections in **(A,C)** were counterstained with hematoxylin. In **(H)**, the brown pigment is the neuromelanin found in SN neurons. Scale bar: **(H)** = 50 μm applies to **(A–G)**, respectively.

Supplementary Figure 2 Box plots showing no difference in the ratio between cresyl violet and H&E stained PC numbers between groups (**A**; Kruskal–Wallis test, *p* > 0.05), even after adjusting for age and gender **(B)**. Conversely, the ratio of SMI-32-ir to H&E PC counts in AD, was significantly lower compared to HC (**C**; Kruskal–Wallis, *p* = 0.03). Adjusting for age and gender yielded similar findings **(D)**. ^*^denotes significant differences between groups.

Supplementary Figure 3 Immunofluorescence images of single labeled Calb (green), Parv (red), and merged (yellow/green) within the cerebellum of a 69-year-old female HC **(A–C)** and a 60-year-old female dementia DS **(D–F)** case. Merged images show CBP dual-labeled PCs (yellow–green) in HC **(C)** and DS **(F)**. Numerous immunofluorescent Parv-ir interneurons were seen in HC **(B)** compared to lesser numbers in DS **(E)**. Immunofluorescence single-labeled Calb (green) **(G)** and SMI-32 (red) **(H)** and merged images of PCs (yellow) **(I)** in a 60-year-old female dementia subject with DS. Note the presence of a rare dendritic torpedo positive for Calb and SMI-32 (white arrows). Scale bars: **(F)** = 50 μm and applies to **(A–E)**; **(I)** = 75 μm and applies to **(G,H)**.

Supplementary Figure 4 Linear regression analysis revealed a significant positive correlation between p75^NTR^-ir PC soma OD values and SMI-32-ir PC counts (**A**; *r* = 0.68; *p* = 0.00038). TrkA-ir PC soma OD values and number correlated positively (**B**; *r* = 0.57; *p* = 0.0053). Significant positive correlations were found between Parv-ir and Calr-ir interneuron counts (**C**; *r* = 0.76; *p* = 0.0000002) and Calr-ir Golgi cell counts (**D**; *r* = 0.74; *p* = 0.0000002) across the three groups. Calr-ir Golgi interneuron number exhibited a strong positive correlation with unipolar brush (**E**; *r* = 0.73; *p* = 0.0000002) and Calr-ir Lugaro cell counts (**F**; *r* = 0.68; *p* = 0.00003).

Supplementary Figure 5 Linear regression analysis revealed a significant negative correlation between Parv- and Calr-ir cell counts and amyloid plaque load across groups. Parv-ir interneuron counts were negatively correlated with Aβ_42_ plaque (**A**; *r* = −0.74; *p* = 0.0000002) and APP/Aβ plaque load (**B**; *r* = −0.61; *p* = 0.00033). Calr-ir cell counts negatively correlated with Aβ_42_ plaque load (**C**; *r* = −0.70; *p* = 0.0000002) and to a lesser extent with APP/Aβ plaque (**D**; *r* = −0.47; *p* = 0.0082) load across groups. Calr-ir Golgi cell counts negatively correlated with Aβ_42_ plaque load (**E**; *r* = −0.71; *p* = 0.0000002) but displayed a weaker association with APP/Aβ plaque load (**F**; *r* = −0.45; *p* = 0.014) across groups.

Supplementary Figure 6 Linear regression analysis revealed a strong significant positive correlation between GL SMI-34-ir torpedo counts and subject age (**A**; *r* = 0.62; *p* = 0.0003) across all groups. Significant negative correlations were seen between NFT Braak scores and counts for SMI-32- (**B**; *r* = −0.66; *p* = 0.00007), p75^NTR^- (**C**; *r* = −0.73; *p* = 0.0000002) and Parv-ir PCs (**D**; *r* = −0.59; *p* = 0.00064). PC TrkA-ir counts (**E**; *r* = −0.57; *p* = 0.001) and p75^NTR^-ir OD values (**F**; *r* = −0.57; *p* = 0.006) correlated negatively with NFT Braak scores across groups.

Supplementary Figure 7 Linear regression analysis revealed a significant negative correlation between NFT Braak scores and TrkA-ir PC soma OD values (**A**; *r* = −0.56; *p* = 0.008) and Calr-ir cell counts (**B**; *r* = −0.58; *p* = 0.00094) across groups.

Supplementary Table 1 Case demographics.
