# Supplementary figures and images for: Cerebellar Calcium-Binding Protein and Neurotrophin Receptor Defects in Down Syndrome and Alzheimer's Disease
Source: Front Aging Neurosci. 2021 Mar 12;13:645334. doi: 10.3389/fnagi.2021.645334 (PMC7994928; doi:10.3389/fnagi.2021.645334)

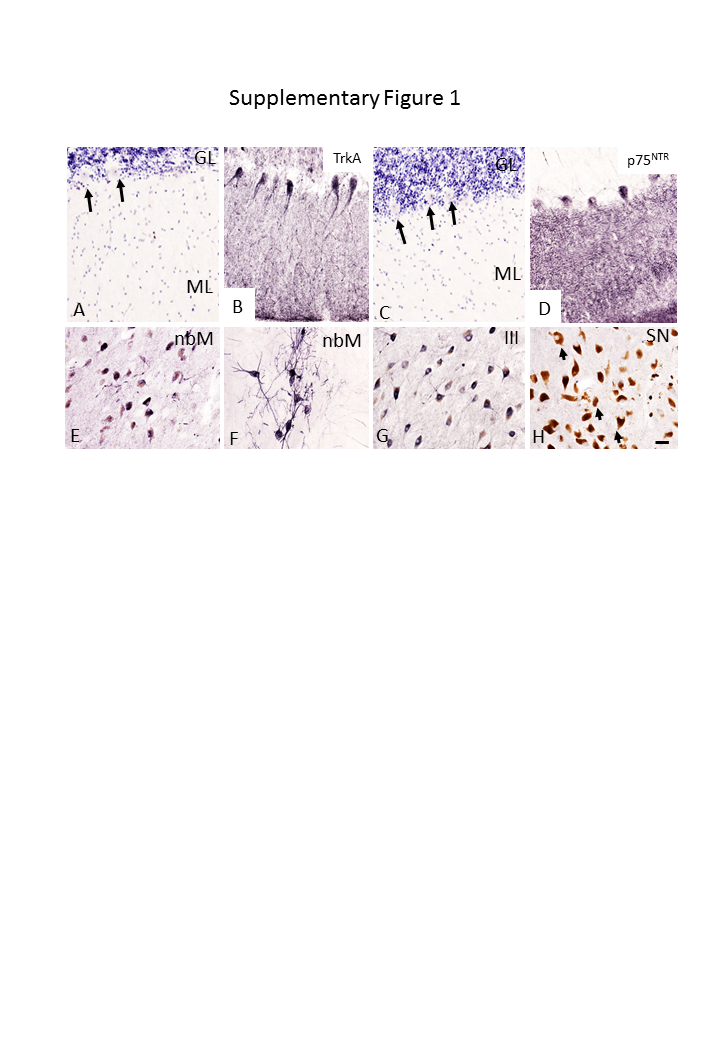

Supplement: Supplementary Figure 1 — Photomicrographs demonstrating the lack of Purkinje cell (PC) TrkA (A, arrows) and p75NTR (C, arrows) immunostaining after omission of each antibody compared to antibody reactiviy for TrkA (B) and p75NTR (D) in a female 47-year-old DSD– case. Lower panels demonstrate that the current TrkA antibody does not immunostain neurons containing TrkB. Similar to previous studies (Mufson and Kordower, 1989; Mufson et al., 1989), cholinergic neurons within the nucleus basalis of Meynert (nbM) are TrkA (E) and p75NTR (F) immunopositive in tissue from a 93-year-old female HC and a 94-year-old female with AD, respectively. (G,H) Images showing TrkA immunopositive (dark blue) neurons in the oculomotor/crainal nerve III (G) in contrast to the absence of TrkA containing neurons in substantia nigra (SN) pars compacta (Sobreviela et al., 1994, H, arrows), which express TrkB, but not TrkA in tissue obtained from a 51-year-old male HC. Findings support the specificity of the TrkA immunostaining of PCs shown in the present study. Sections in (A,C) were counterstained with hematoxylin. In (H), the brown pigment is the neuromelanin found in SN neurons. Scale bar: (H) = 50 μm applies to (A–G), respectively. [file Image_1.TIF]

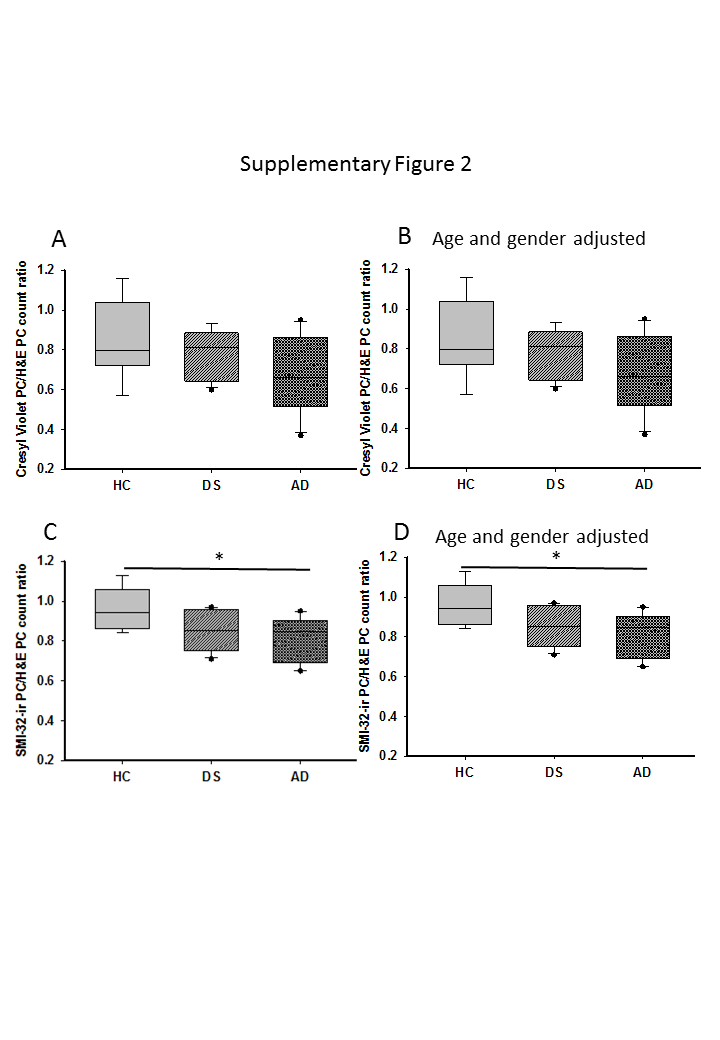

Supplement: Supplementary Figure 2 — Box plots showing no difference in the ratio between cresyl violet and H&E stained PC numbers between groups (A; Kruskal–Wallis test, p > 0.05), even after adjusting for age and gender (B). Conversely, the ratio of SMI-32-ir to H&E PC counts in AD, was significantly lower compared to HC (C; Kruskal–Wallis, p = 0.03). Adjusting for age and gender yielded similar findings (D). *denotes significant differences between groups. [file Image_2.TIF]

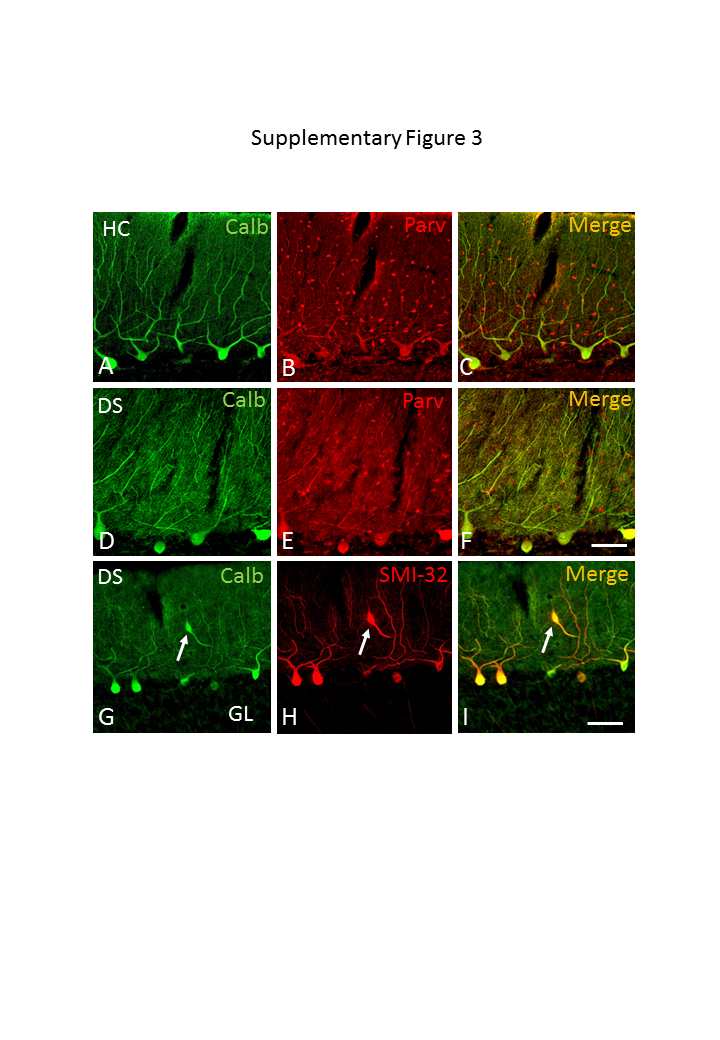

Supplement: Supplementary Figure 3 — Immunofluorescence images of single labeled Calb (green), Parv (red), and merged (yellow/green) within the cerebellum of a 69-year-old female HC (A–C) and a 60-year-old female dementia DS (D–F) case. Merged images show CBP dual-labeled PCs (yellow–green) in HC (C) and DS (F). Numerous immunofluorescent Parv-ir interneurons were seen in HC (B) compared to lesser numbers in DS (E). Immunofluorescence single-labeled Calb (green) (G) and SMI-32 (red) (H) and merged images of PCs (yellow) (I) in a 60-year-old female dementia subject with DS. Note the presence of a rare dendritic torpedo positive for Calb and SMI-32 (white arrows). Scale bars: (F) = 50 μm and applies to (A–E); (I) = 75 μm and applies to (G,H). [file Image_3.TIF]

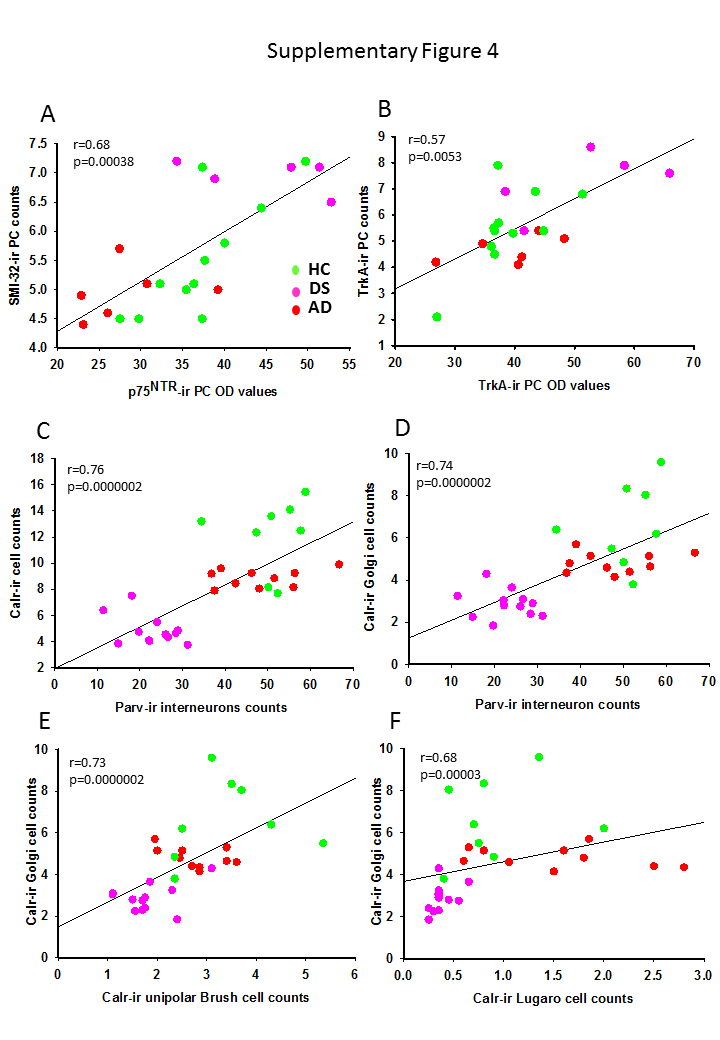

Supplement: Supplementary Figure 4 — Linear regression analysis revealed a significant positive correlation between p75NTR-ir PC soma OD values and SMI-32-ir PC counts (A; r = 0.68; p = 0.00038). TrkA-ir PC soma OD values and number correlated positively (B; r = 0.57; p = 0.0053). Significant positive correlations were found between Parv-ir and Calr-ir interneuron counts (C; r = 0.76; p = 0.0000002) and Calr-ir Golgi cell counts (D; r = 0.74; p = 0.0000002) across the three groups. Calr-ir Golgi interneuron number exhibited a strong positive correlation with unipolar brush (E; r = 0.73; p = 0.0000002) and Calr-ir Lugaro cell counts (F; r = 0.68; p = 0.00003). [file Image_4.TIF]

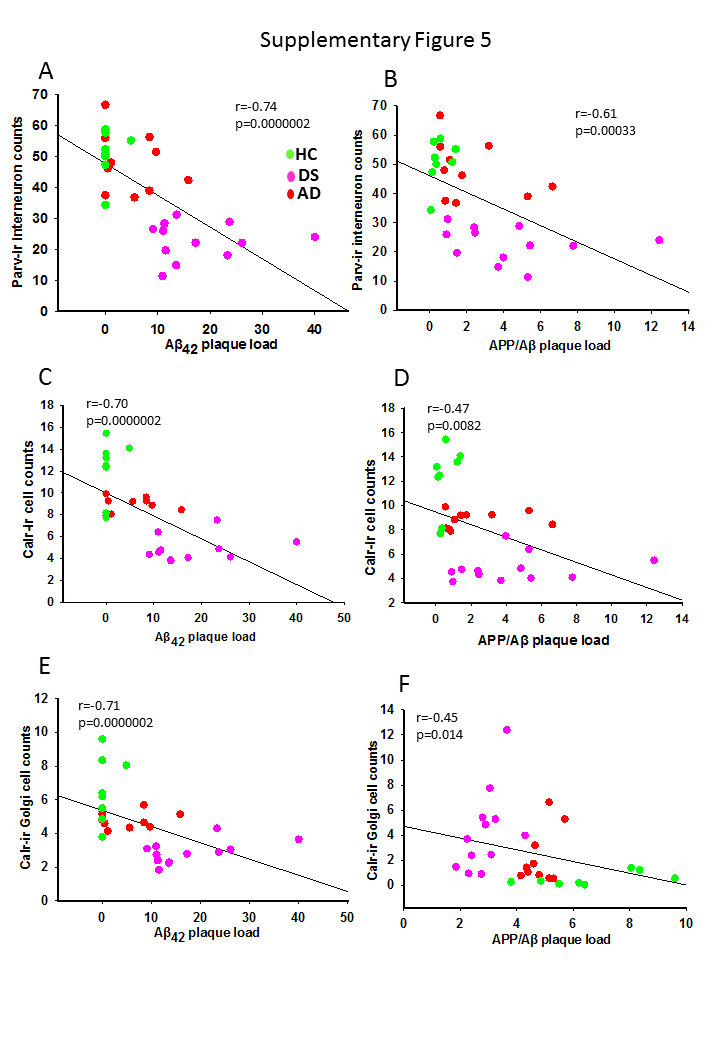

Supplement: Supplementary Figure 5 — Linear regression analysis revealed a significant negative correlation between Parv- and Calr-ir cell counts and amyloid plaque load across groups. Parv-ir interneuron counts were negatively correlated with Aβ42 plaque (A; r = −0.74; p = 0.0000002) and APP/Aβ plaque load (B; r = −0.61; p = 0.00033). Calr-ir cell counts negatively correlated with Aβ42 plaque load (C; r = −0.70; p = 0.0000002) and to a lesser extent with APP/Aβ plaque (D; r = −0.47; p = 0.0082) load across groups. Calr-ir Golgi cell counts negatively correlated with Aβ42 plaque load (E; r = −0.71; p = 0.0000002) but displayed a weaker association with APP/Aβ plaque load (F; r = −0.45; p = 0.014) across groups. [file Image_5.tif]

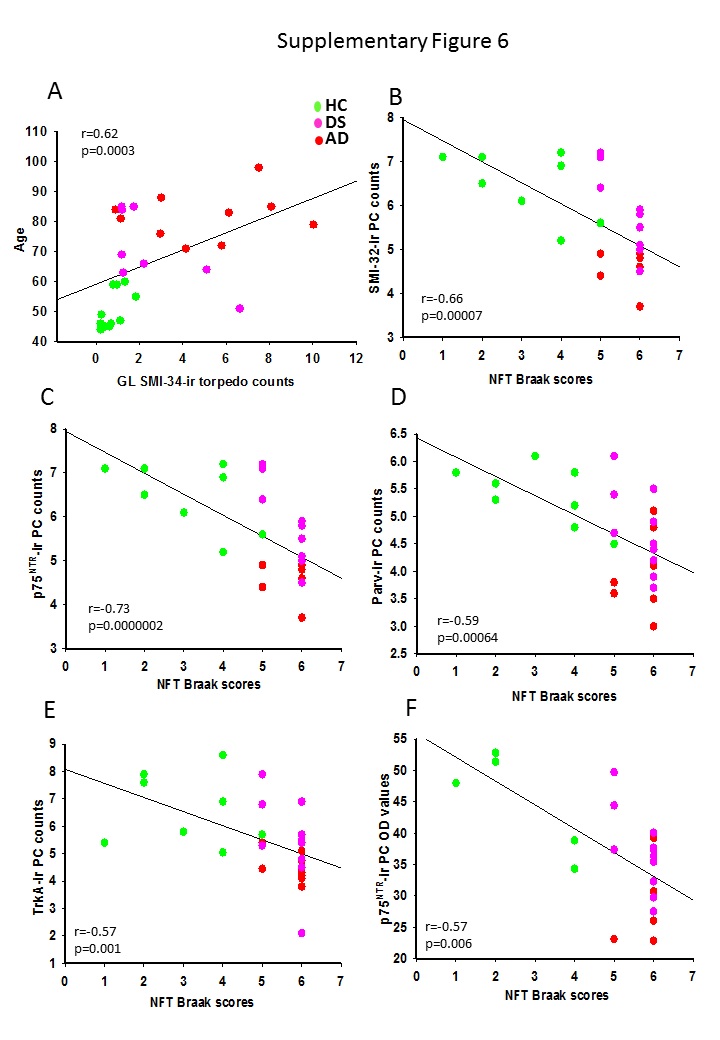

Supplement: Supplementary Figure 6 — Linear regression analysis revealed a strong significant positive correlation between GL SMI-34-ir torpedo counts and subject age (A; r = 0.62; p = 0.0003) across all groups. Significant negative correlations were seen between NFT Braak scores and counts for SMI-32- (B; r = −0.66; p = 0.00007), p75NTR- (C; r = −0.73; p = 0.0000002) and Parv-ir PCs (D; r = −0.59; p = 0.00064). PC TrkA-ir counts (E; r = −0.57; p = 0.001) and p75NTR-ir OD values (F; r = −0.57; p = 0.006) correlated negatively with NFT Braak scores across groups. [file Image_6.tif]

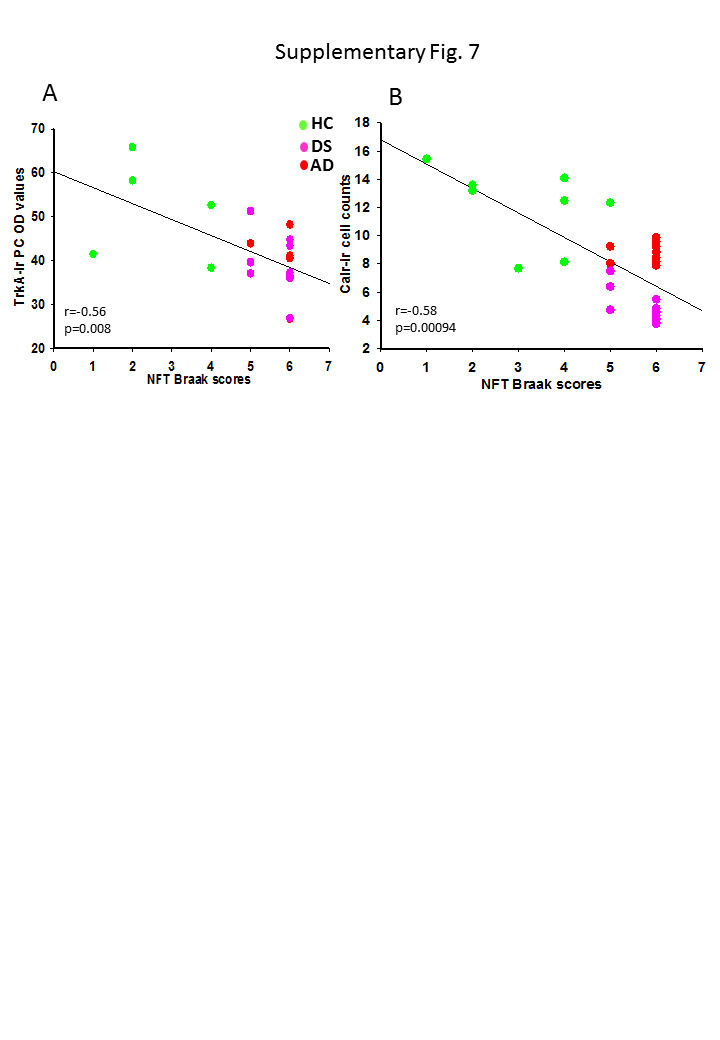

Supplement: Supplementary Figure 7 — Linear regression analysis revealed a significant negative correlation between NFT Braak scores and TrkA-ir PC soma OD values (A; r = −0.56; p = 0.008) and Calr-ir cell counts (B; r = −0.58; p = 0.00094) across groups. [file Image_7.tif]
