# Supplementary material for: Cerebellar Calcium-Binding Protein and Neurotrophin Receptor Defects in Down Syndrome and Alzheimer's Disease
Source: Front Aging Neurosci. 2021 Mar 12;13:645334. doi: 10.3389/fnagi.2021.645334 (PMC7994928; doi:10.3389/fnagi.2021.645334)
Supplement: Supplementary Table 1 — Case demographics. [file Table_1.DOCX]

**Supplementary Table 1. Case Demographics**

| **Case ID** | **Gender** | **Age (y)** | **Clinical Diagnosis^a^** | **PMI (h)** | **Brain Weight (g)** | **Braak Score** | **Tissue Source^b^** |
| --- | --- | --- | --- | --- | --- | --- | --- |
| 1 | M | 51 | HC | 11.5 | 1260 | I | RU |
| 2 | M | 63 | HC | 13.0 | 1275 | II | RU |
| 3 | F | 64 | HC | 20.0 | 1400 | III | RU |
| 4 | F | 66 | HC | 17.0 | 1300 | II | RU |
| 5 | F | 69 | HC | 11.5 | 1200 | IV | RU |
| 6 | F | 84 | HC | 18.0 | 1200 | IV | RU |
| 7 | M | 85 | HC | 17.0 | 1260 | V | RU |
| 8 | F | 85 | HC | 3.0 | 1180 | IV | RU |
| 9 | F | 71 | AD | 4.0 | 925 | VI | RU |
| 10 | F | 72 | AD | 3.5 | 1045 | VI | RU |
| 11 | F | 76 | AD | 5.0 | 970 | VI | RU |
| 12 | F | 79 | AD | 4.0 | 995 | NA | RU |
| 13 | M | 81 | AD | 4.5 | 1350 | VI | RU |
| 14 | F | 83 | AD | 6.0 | 1000 | V | RU |
| 15 | M | 84 | AD | 7.0 | 1430 | V | RU |
| 16 | F | 85 | AD | 6.0 | 1070 | VI | RU |
| 17 | M | 88 | AD | 6.5 | 1280 | VI | RU |
| 18 | F | 98 | AD | 5.5 | 1020 | VI | RU |
| 19 | F | 44 | DS | 8.0 | 1060 | V | RU |
| 20 | F | 45 | DS | 2.8 | NA | VI | UCI ADRC |
| 21 | F | 45 | DS | 3.2 | NA | VI | UCI ADRC |
| 22 | M | 46 | DS | 6.4 | NA | VI | UCI ADRC |
| 23 | M | 46 | DS | 20.0 | 1090 | VI | RU |
| 24 | F | 47 | DS | 5.0 | NA | V | RADC |
| 25 | M | 49 | DS | 2.2 | NA | VI | UCI ADRC |
| 26 | M | 55 | DS | 4.5 | NA | VI | UCI ADRC |
| 27 | F | 57 | DS | 5.3 | NA | VI | UCI ADRC |
| 28 | F | 59 | DS | 4.5 | NA | VI | RU |
| 29 | F | 59 | DS | 13.0 | 700 | VI | BNI |
| 30 | F | 60 | DS | 15.0 | 1030 | V | RU |

^a^HC: Healthy Control; AD: Alzheimer’s Disease; DS: Down Syndrome; NA: not available

^b^RU: Rush University Department of Pathology; UCI ADRC: University of California at Irvine Alzheimer’s Disease Research Center; BNI: Barrow Neurological Institute at St. Joseph’s Hospital and Medical Center
